# Low-Friction Soft Robots for Targeted Bacterial Infection Treatment in Gastrointestinal Tract

**DOI:** 10.34133/cbsystems.0138

**Published:** 2024-07-05

**Authors:** Ben Wang, Yunrui Chen, Zhicheng Ye, Haidong Yu, Kai Fung Chan, Tiantian Xu, Zhiguang Guo, Weimin Liu, Li Zhang

**Affiliations:** ^1^ College of Chemistry and Environmental Engineering, Shenzhen University, Shenzhen 518060, China.; ^2^ Guangxi Key Laboratory of Processing for Non-Ferrous Metals and Featured Materials, School of Resource, Environments and Materials, Guangxi University, Nanning 530004, China; ^3^ Chow Yuk Ho Technology Centre for Innovative Medicine, The Chinese University of Hong Kong, Shatin, New Territories, Hong Kong SAR, China.; ^4^ Guangdong Provincial Key Laboratory of Robotics and Intelligent System, Shenzhen Institute of Advanced Technology, Chinese Academy of Sciences, Shenzhen 518055, China.; ^5^ Key Laboratory of Biomedical Imaging Science and System, Shenzhen Institute of Advanced Technology, Chinese Academy of Sciences, Shenzhen 518055, China.; ^6^ Hubei Collaborative Innovation Centre for Advanced Organic Chemical Materials and Ministry of Education Key Laboratory for the Green Preparation and Application of Functional Materials, Hubei University, Wuhan 430062, China.; ^7^ State Key Laboratory of Solid Lubrication, Lanzhou Institute of Chemical Physics, Chinese Academy of Science, Lanzhou 730000, China.; ^8^ Department of Mechanical and Automation Engineering, The Chinese University of Hong Kong, Shatin, New Territories, Hong Kong SAR, China.; ^9^ Multi-Scale Medical Robotics Center, Hong Kong Science Park, Shatin, New Territories, Hong Kong SAR, China.

## Abstract

Untethered and self-transformable miniature robots are capable of performing reconfigurable deformation and on-demand locomotion, which aid the traversal toward various lumens, and bring revolutionary changes for targeted delivery in gastrointestinal (GI) tract. However, the viscous non-Newtonian liquid environment and plicae gastricae obstacles severely hamper high-precision actuation and payload delivery. Here, we developed a low-friction soft robot by assembly of densely arranged cone structures and grafting of hydrophobic monolayers. The magnetic orientation encoded robot can move in multiple modes, with a substantially reduced drag, terrain adaptability, and improved motion velocity across the non-Newtonian liquids. Notably, the robot stiffness can be reversibly controlled with magnetically induced hardening, enabling on-site scratching and destruction of antibiotic-ineradicable polymeric matrix in biofilms with a low-frequency magnetic field. Furthermore, the magnetocaloric effect can be utilized to eradicate the bacteria by magnetocaloric effect under high-frequency alternating field. To verify the potential applications inside the body, the clinical imaging-guided actuation platforms were developed for vision-based control and delivery of the robots. The developed low-friction robots and clinical imaging-guided actuation platforms show their high potential to perform bacterial infection therapy in various lumens inside the body.

## Introduction

Traditional medical tools and robots struggle to access various lumens and cavities inside the human body [1–3]. However, robots have successfully been implemented in hazardous, harmful, unpleasant, and inaccessible environments. The use of miniature, untethered, and self-transformable robots for targeted delivery and therapy in the digestive system has the potential to revolutionize medical applications, especially since navigating the small and hard-to-reach lumens within the digestive system poses substantial challenges [4–16]. Traditional endoscopic methods, such as gastroscopy and colonoscopy, can cause discomfort and pose risks for patients due to the need for sedation [17,18]. Due to the highly invasive, mucosa-damaging, and costly nature of current mainstream treatment methods, there is a pressing need for more comfortable, painless, and benign diagnostic, therapeutic, and theragnostic methods. In comparison to other organs, the digestive system has a weaker immune response, making it possible to use a wider variety of carrier materials. Researchers have been experimenting with a range of microrobotic systems and strategies to enable the remote delivery of microrobots and payloads to the stomach and intestine, taking local environment into careful consideration [19–26]. However, significant challenges remain in the digestive system. For example, intestinal peristalsis and highly viscous mucus pose major obstacles to the effective actuation and control of miniature robots within these cavities [27,28], while surface wrinkles also impede the motion of microrobots along the GI tract [29]. Therefore, researchers need to figure out optimized strategies to design miniature robots with substantially reduced surface friction and terrain adaptability, and develop integrated system/platform to enable the real-time tracking of microrobots with adequate signal-to-noise ratios inside the body [30–35].

Legs are common among a variety of land-dwelling animals and insects. These appendages lift the body off the ground, reducing friction with the surface and allowing for greater freedom of movement, even over obstacles. This mode of locomotion also requires less energy than wriggling and scuttling [36–39]. Therefore, animals/insects with legs are better suited to navigating complex terrains, such as centipedes and millipedes that have more than dozens of legs. The large number of legs offers enlightening role models to construct soft robots with multiple legs, which could prove a highly effective solution for navigating the complicated terrain of the gastrointestinal (GI) tract while minimizing surface adhesion. In recent years, magnetically driven robots designed by mimicking natural biological structures and behaviors have fascinated robotics scientists with their advantages of easy access, biosecurity, and ease of programming [40–43].

Apart from the robot structure, surface chemistry is also an important factor that may affect the motion friction in aqueous environment. Water comprises polar triatomic molecules with a bond angle of 104.5°. Surfaces with a strong polarity favor the capture of water molecules, whereas surfaces featuring nonpolar surface termination favor adverse effects [44]. Robot surface polarity is strongly associated with the surface terminal groups. In nature, various species can reduce drag forces through rational design of the surface properties. For instance, water striders stand and skate freely on water due to the oriented microsetae and fine nanogrooves [45]. Earthworms squirm in soil without injuring their surface tissues because the dynamically secreted mucus on their surface reduces motion friction [46]. Therefore, surface modification and structuring are promising means to minimize the surface adhesion and friction between tiny robots and their surroundings [47–49].

To improve the ability of miniature robots to navigate through the GI tract with the non-Newtonian liquid environment and plicae gastricae obstacles, we developed a low-friction soft robot (LFSR) with exceptional stretchability and softness akin to human tissue, thereby eliminating the potential for tissue damage during targeted delivery through the GI tract. Due to the modular design of the soft robots with predesigned magnetic orientation, the soft robots can move in multiple modes, with a substantially reduced drag and improved motion velocity across the non-Newtonian liquids, such as mucus environment inside GI tract. The ultra-low surface friction and adhesion are generated by the rational design of the soft robots with multiple cone structures and hydrophobic decoration. Notably, the robot stiffness can be reversibly controlled with magnetically induced hardening, enabling on-demand actuation and removal of bacterial films by on-site scratching with a low-frequency magnetic field. Furthermore, the magnetocaloric effect can be utilized to heat up the LFSRs through a high-frequency alternating magnetic field (AMF), enabling the thorough eradication of the bacteria. To verify the potential use of the LFSRs inside the body, we developed clinical imaging-guided actuation platforms that integrate the clinical imaging modalities, i.e., x-ray imaging and ultrasound (US) imaging, together with a robot arm equipped magnetic actuation setup. The robot’s motion trajectory, as well as the biofilm scratching, can be easily detected using clinical imaging-guided actuation platforms. The developed low-friction robots and the clinical imaging-guided actuation platforms show their high potential to perform bacterial infection therapy in various non-Newtonian liquid lumens.

## Materials and Methods

### Materials

NdFeB magnetic microparticles (37 μm) were purchased from Guangzhou New Nord Transmission Parts Co. Ltd. Fe_3_O_4_ magnetite nanoparticles (30 μm) were purchased from Shanghai Chengyue Flagship Store. Ecoflex elastomer was purchased from Smooth-On. The hole punch and molds were purchased from Xinxin Personality Workshop Co. Ltd.

### Preparation of the LFSR

#### Fabrication of the magnetic soft foot layer of the multi-legged soft robot

Ecoflex A prepolymer and B prepolymer were mixed evenly. Then, the mixed Ecoflex prepolymer was degassed in a desiccator. Afterward, an appropriate amount of Ecoflex prepolymer and Fe_3_O_4_ magnetic microparticles were balanced and stirred to prepare a magnetic prepolymer. The weight ratio of Ecoflex prepolymer mixture and Fe_3_O_4_ magnetic microparticles was 2:1. Then, the mixture was evenly distributed on a polyethylene glycol terephthalate (PET) sheet by spin coating. A cylindrical NdFeB permanent magnet (diameter 44 mm, height 28 mm) was placed under the plate, and the external magnetic field originating from the magnet was applied to generate tapered cylindrical (cone) feet. The distance between the magnet and the plate ranges from 5 to 40 mm, and the magnetic field strength decays with distance. After leg formation, the PET plate was placed into an oven for curing (15 min, 60 °C), followed by peeling the finished film off the plate and cutting into rectangular shapes to obtain a multi-legged layer composed of magnetic particle.

#### Fabrication of the magnetic soft body layer of the multi-legged soft robot

The magnetic soft body layer is prepared through a similar method to the soft foot layer, with the Fe_3_O_4_ particles substituted by the NdFeB particles. The PET sheet was then placed in an oven for curing (15 min, 60 °C). The resulting finished film (thickness approximately 0.5 mm) was cut into robot body parts of different geometries using a punch, with a circular mold diameter of 4 mm and a rectangular mold of 5 mm long and 3.5 mm wide, and then placed in the electromagnet magnetic field-generating device for magnetization programming.

#### Magnetizing process of the magnetic soft body layer

Body components of different geometries are fixed using auxiliary fixtures, and magnetization programming is performed using a vibrating sample magnetometer (VSM; EZ7, MicroSense). The strength of the magnetizing field is 0.5 T. The degree of magnetization is related to the concentration of the component NdFeB particles and the strength of the magnetization field. The VSM generates a magnetic field in the gap between its 2 parallel circular plates. The direction of the magnetic field is perpendicular to the circular plate, and its strength is controlled by its ends. The body components are fixed to their corresponding orientations in a jig, and the jig is fixed to a 5-cm-wide cubic acrylic sheet. This cubic acrylic slab is sandwiched between 2 circular slabs of the VSM, placing the body component at the geometric center of the gap. A magnetic field is then generated for strengthening.

### LFSR assembly procedure

The LFSR is assembled by combining various geometrically shaped soft layers and connecting them to the multi-legged layer using an appropriate adhesive. The selected adhesive undergoes curing upon contact with neighboring body parts, allowing for a secure bond. The assembly takes place in a controlled laboratory setting where auxiliary fixtures are utilized to position and align the components, resulting in the desired three-dimensional geometry and magnetization profile of the robot. This mechanical fixation method effectively mitigates magnetic interactions between different body parts, preventing any potential collapses or dislodgement during the manufacturing phase.

To carry out the assembly, precision tweezers (5-SA Outils Rubis SA, Switzerland) serve as the primary tools, and toothpicks are employed for adhesive application. When manipulating the components with tweezers, it is recommended to handle only one tweezer at a time while using the other tweezer (held by the opposite hand) to stabilize the fixture or the assembled sections of the robot. This approach simplifies the majority of the movements, predominantly relying on translational motion, thereby facilitating the overall process.

### Mechanical testing

After the curing process, the soft foot layer and soft body layer of the robot were cut into dog bone-shaped specimens of known dimensions (width of 2 mm and standard length of 35 mm) for tensile testing. The cross-sectional area of each specimen was calculated by measuring the thickness and length. The specimens were tested on a mechanical testing machine (MTS\SANS CMT2000) using a 50-N load cell and a tensile rate of 10 mm/min. Nominal stress–strain curves for the 2 materials were plotted, and fitting of the experimental curves was performed using the neo-Hookean model to obtain the shear modulus.

### LFSR sterilization through magnetic hyperthermia

#### Culturing of bacterial biofilm

A single colony of *S. aureus* was picked and added to 3 ml of brain heart infusion (BHI) broth medium. The mixture was then incubated on a shaking incubator (Bluepard THZ-103B) at a constant temperature of 37 °C for 16 h. After centrifugation using a centrifuge (Cen Lee 16K) and subsequent washing with physiological saline solution, the bacterial pellet was resuspended in 6 ml of BHI medium containing 2% glucose. The suspension was thoroughly mixed, and 2 ml of the bacterial culture was distributed onto a confocal culture dish. The dish was incubated at 37 °C in a temperature-controlled incubator (Faithful DH45L) for 48 h.

#### Biofilm removal and sterilization

The soft robot was placed inside a culture dish containing the biofilm and a small amount of physiological saline solution. The robot was then magnetically driven to scrape off the biofilm, resulting in a suspension. Subsequently, the suspension, along with the robot, was placed under a magnetic induction heating device (GUF 500K-1, Shenzhen Polar Magnetic Induction Technology Co. Ltd.) for magnetic hyperthermia sterilization. The temperature of the suspension was monitored using a thermal imaging camera (FLUKE VT104A) to ensure that it remained above 50 °C for 10 min.

#### Bacterial fluorescence staining

The bacterial viability assay kit (AAT Bioquest) was used for fluorescence measurement. Two microliters of the staining reagent was added to 200 μl of physiological saline solution and mixed thoroughly in the dark. Then, 50 μl of bacterial culture was added to the staining solution, mixed well, and incubated in the dark for 15 min. The stained solution was pipetted onto a glass slide, covered with a coverslip, and observed under a high-resolution fluorescence microscope (Soptop, Ningbo Sunyu Instrument Co. Ltd.) equipped with a 40× objective lens. Fluorescence emitted by live bacteria with intact cell membranes was excited by red light, resulting in green fluorescence. In contrast, dying or dead bacteria emitted red fluorescence when excited by blue light.

#### Biofilm colony plate culture

Bacterial culture (100 μl) was transferred using a micropipette and added to 900 μl of physiological saline solution. The mixture was thoroughly mixed, and 10 μl of the diluted bacterial suspension was evenly spread onto solid BHI medium. The plate was then incubated at 37 °C for 16 h for bacterial colony counting.

#### Gastric mucosal biofilm culture

A clean pig stomach was spread out in a culture dish, forming a concave shape. Then, 10 ml of the abovementioned bacterial culture was added to the gastric mucosa, and the dish was covered with another dish lid to maintain the position of the pig stomach. The culture dish was then incubated at 37 °C for 24 h.

## Results and Discussion

### System design

**Fig. 1.**
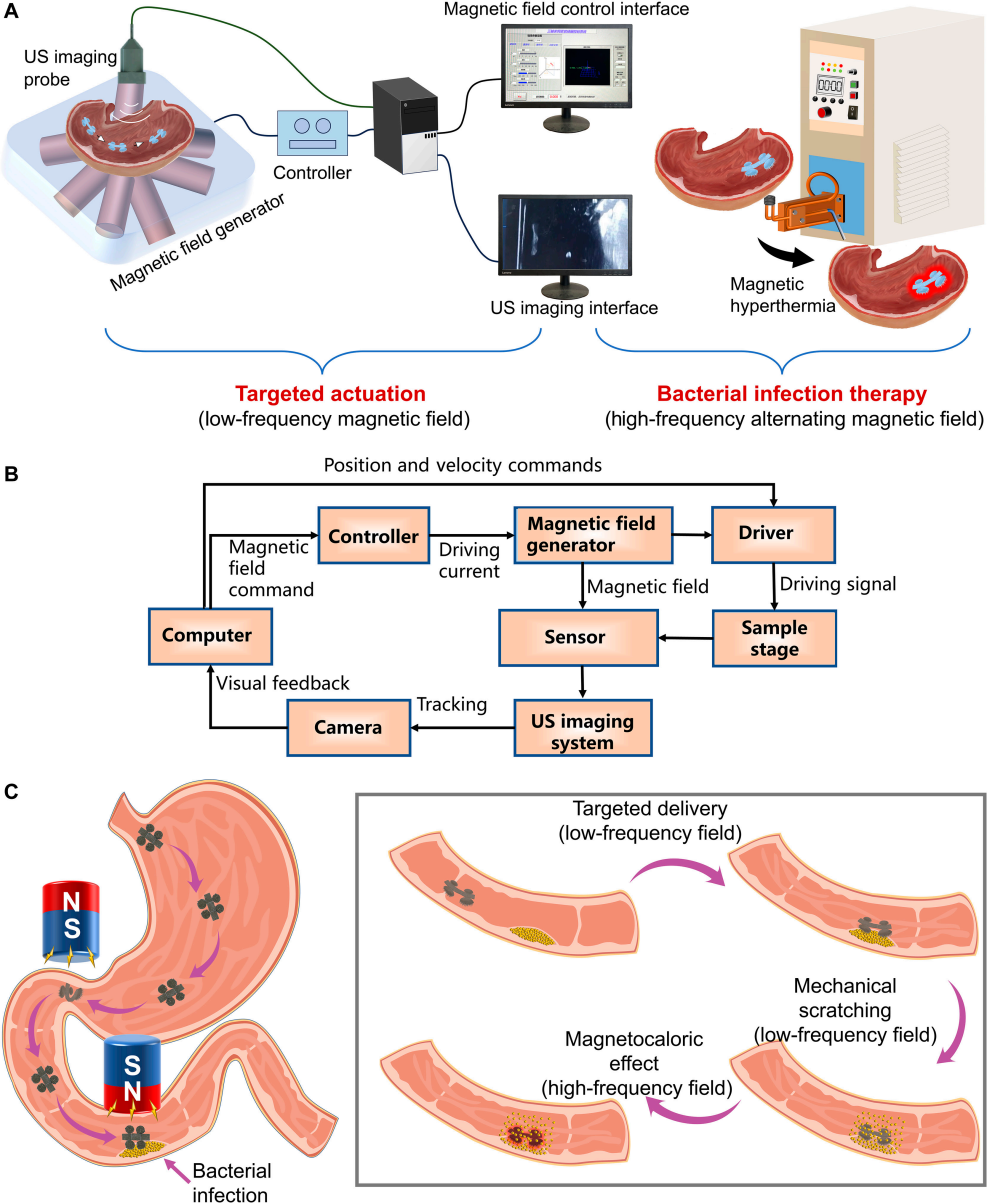
Combination of magnetic field generator, US imaging, and low-friction soft microrobots for targeted therapy in GI tract. (A) Schematic showing the integrated system for targeted therapy in GI tract. (B) Schematic showing the control and imaging logic during guided actuation of soft robots. (C) Schematic showing the targeted delivery, mechanical scratching, and magnetocaloric effect of the low-frequency soft robots for treatment of bacterial infection.

We developed a system that combined the magnetic field generator, robot arm, US imaging, and the LFSRs together to achieve the imaging-guided delivery of the LFSRs to the deep lesion site for targeted therapy of bacterial infection (Fig. 1A). Legged insects, such as centipedes with plenty of tiny soft legs, offer enlightening role models to construct soft robots with reduced surface adhesion and friction to the ground (Fig. S1). Inspired by centipedes, the LFSRs are equipped with 4 circular legs, and each of the legs is assembled with densely arranged soft cone structures. Both the body part and foot part of the robot can be encoded with specific magnetic orientation by embedding of NdFeB particles so that each part can respond to the external magnetic field separately, generating on-demand shape deformation and motion modality. To achieve efficient locomotion in mucus environment of GI tract, the soft robots are grafted with a hydrophobic monolayer to realize a substantially reduced interfacial drag. The bacterial infection therapy is achieved by a synergy of mechanical scratching under low-frequency magnetic field and hyperthermia under high-frequency AMF, getting rid of the usage of antibacterial drugs. At a low-frequency magnetic field, the mechanical stiffness of the soft cones of the robots can be significantly enhanced by magnetically induced hardening effect, resulting in an effective scratching of the surface biofilms. Bacterial biofilm represents a sophisticated architectural arrangement developed by bacterial communities to facilitate adaptation to challenging environmental circumstances, including conditions characterized by low pH and elevated temperature. Upon disruption of the biofilm matrix, bacteria are deprived of their protective enclave, rendering them susceptible to heightened temperatures, thereby increasing their vulnerability to extermination. Furthermore, the high-frequency AMF can cause the magnetization reversal process periodically. During the process that the magnetic particles inside the robot come back to their relaxed states, thermal energy is produced and the temperature is increased. The raised temperature triggers the death of the bacteria. The targeted actuation of the LFSRs can be well tracked by using medical imaging tools, i.e., US imaging and x-ray imaging. Figure 1B gives the control and imaging logic during guided actuation of soft robots. The targeted bacterial infection therapy can be divided into 3 steps. The first step is the targeted delivery of the robots toward the lesion location with a low-frequency magnetic field; the second step is the mechanical scratching of the bacterial films on the lumen wall with a low-frequency magnetic field; the final step is on-site magnetic hyperthermia to kill the bacteria with a high-frequency magnetic field (Fig. 1C). It is remarkable that although the developed LFSRs are proposed for the treatment of bacterial infection in GI tract, they may also be applicable for the other kind of lumens with mucus layers inside the body.

### Preparation of LFSRs

**Fig. 2.**
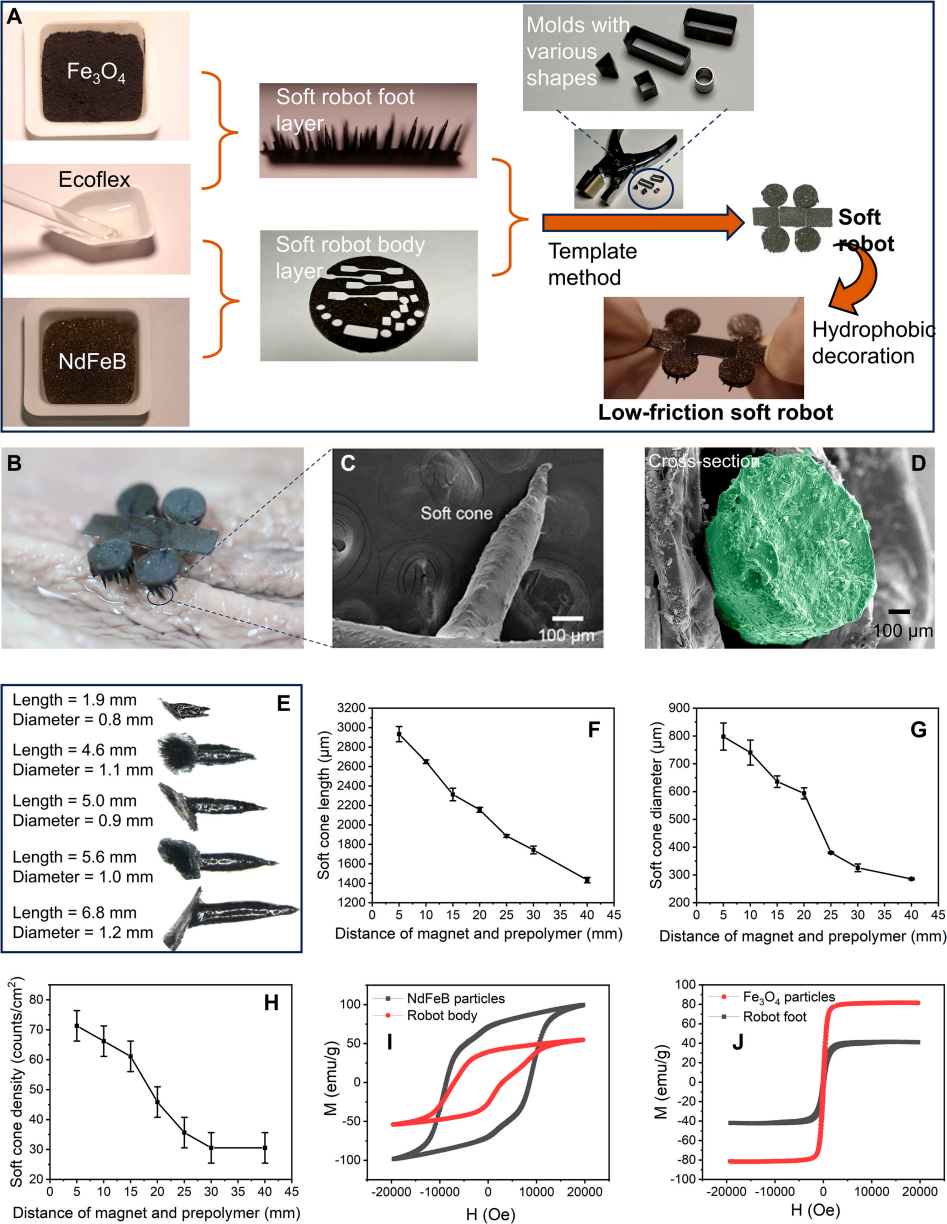
Fabrication of the LFSRs with hydrophobic decoration. (A) Photographs showing the raw materials and the fabrication procedure of the LFSRs. (B) Optical images of an LFSR. (C and D) SEM micrograph of a single foot and its cross-section. (E) Optical microscopy of the cone length of LFSRs with various lengths and diameters. (F) Dependence of the soft cone length on the distance of magnet and prepolymer (mixture of Fe_3_O_4_ particles and Ecoflex). (G) Dependence of the soft cone diameter on the distance of magnet and prepolymer. (H) Dependence of the soft cone density on the distance of magnet and prepolymer. (I) Hysteresis loops (applied magnetic field strength on the magnetic moment) of NdFeB particles and the soft body layer of the LFSR. (J) Hysteresis loops of Fe_3_O_4_ particles and the soft robot foot layer of the LFSR.

The LFSRs are prepared through a multi-modular assembly. The fabrication contains 2 steps: i.e., fabrication of the soft robot body layer and the robot foot layer (Fig. 2A and Fig. S2). The preparation of the magnetic soft foot layer of the multi-legged soft robot was conducted by mixing elastomers and magnetic Fe_3_O_4_ particles at a mass ratio ranging from 1:1 to 2:1. After drawing with a permanent magnet, cone-shaped feet were observed under an optical microscope. The magnetic nanoparticle was evenly dispersed in the Ecoflex cones, and the cones (feet) were evenly dispersed on the foot layer, which is desirable for enhancing the maneuverability and adaptability of the robots to the wrinkled structures in the GI tract. The inclusion of magnetic particles increased the roughness of the feet, thereby decreasing the contact area and friction of the robot with the surface of the GI tract during its crawling motion. The magnetic soft body layer is prepared through a similar method to the soft foot layer, with the Fe_3_O_4_ particles substituted by the NdFeB particles. After that, both the soft foot layer and body layer can be processed into various shapes and sizes by selecting specific molds. Then, these modules can be assembled with ease by using prepolymer of Ecoflex as the glue. The assembled soft robots are finally decorated with hydrophobic silane to generate the LFSRs. The paddle-crawling motion of an LFSR under a rotating magnetic field is shown in Fig. S3. The walking gait of the robot was programmed by patterning alternating separate magnetization within the legs. We show the design and the locomotion mechanism of an LFSR robot in Fig. S3. The legs of the LFSR robot bear alternating magnetization. By using the stroke action under a rotating magnetic field, the robot can stand on legs with its body up and move forward. The arms move forward alternately with the legs kicking the ground throughout the stroke. Ground friction and fluid resistance combine to generate propulsion. Friction-based propulsion was dominant when the robot sank to the bottom of the liquid, and the cone structure on the legs could increase the frictional propulsion force. Figure 2B and Fig. S4 show the typical photograph of an LFSR. The scanning electron microscopy (SEM) images in Fig. 2C and D indicate that the robots contain shape cone-like structures on their feet. The length and diameter of the cone structures of the LFSRs can be controlled with a wide range by simply adjusting the distance between a magnet and the prepolymer (mixture of Fe_3_O_4_ particles and Ecoflex). The soft cones can also be generated using other elastomers, like polydimethylsiloxane (PDMS), and other magnetic particles, such as NdFeB particles, to endow them with an increase modulus (Fig. S5). As shown in Fig. 2E to H and Fig. S6, the cone length and diameter decrease with the increase of the distance between a magnet and the prepolymer. The splitting occurs because of the attractive forces from the dipole generated by the magnetic field, which leads to a morphological change of the liquid surface due to the formation of particle chains within the liquid. This splitting is caused by a combination of the high magnetic field strength *H* and steep vertical magnetic field gradient *dH*/*dz*. The characteristic size *D* of the mixture is much larger than the critical wavelength of the Rosensweig instability λ_C_, resulting in the formation of numerous vertically aligned, cone-structured droplets (Fig. S7) [50].

$$D>\lambda_{C}\approx 2\pi \sqrt{\frac{\sigma }{\frac{d}{\mathrm{dz}}\left(\mu_{0}\mathrm{HM}\right)}}$$

where σ represents the surface tension of the liquid, μ_0_ signifies the permeability of vacuum, and *M* denotes the magnetization. Within this experiment, the magnetic liquid mixture exhibits susceptibility to splitting, resulting in split cone-structured feet whose length and diameter can be manipulated by altering the vertical distance between the magnet and prepolymer. The cone density (counts of the cones per centimeter) decreases with the increase of the distance between a magnet and the prepolymer. The relationship between the magnetic field strength and the distances is shown in Fig. S8. The attenuation rate of the magnetic field strength is very fast as the distance increases. When the distance reaches 20 cm, the magnetic field strength is approximately 0. A densely arranged soft cones facilitate the motion stability of the LFSRs. We also measured the magnetic properties of the soft body and soft foot parts. The soft body is permanently magnetic with a considerable remanence (Fig. 2I), denoting that the body part can be programmed and encoded with different magnetic orientations at different regions. The soft feet are superparamagnetic with a saturation magnetization of ~40 emu/g (Fig. 2J). The soft feet will become demagnetized as soon as the external magnetic field is removed. The definitely different magnetism will endow the soft robots with different kinds of shape deformation at their body part and foot part. To examine the multilegged soft robot’s suitability of movement in GI tract, especially in the stomach with a strong acidic liquid, the soft robot is immersed in an acidic solution (pH 1) and the result shows that the robot structure remained steadfastly unchanged with exceptional corrosion resistance (Fig. S9). The LFSR also shows similar motion performance before (0.21 cm/s) and after the acid leaching (0.20 cm/s).

The LFSRs show exceptional softness in both the body part and the foot part. Figure S10A shows the stretchability of the LFSRs. While the robot body part and the foot part are placed onto a tensile machine, one can observe that both exhibit excellent stretchability (Fig. S10B and Movie S1). The stress–strain curve in Fig. S10C indicates that the body part and foot part of the soft robot show comparable elastic moduli of approximately 142 and 146 kPa, respectively, through substitution into the *E* = σ/ε formula (Fig. S10C). When compared with the human tissues and organs (Fig. S10D), one can observe that the modulus of the soft robots is substantially lower than that of cartilage. This feature ensures that the robot moves softly through such soft tissues and will mitigate damage, providing a secure foundation for in vivo drug delivery.

### Motion performance of soft robots with different wettabilities

**Fig. 3.**
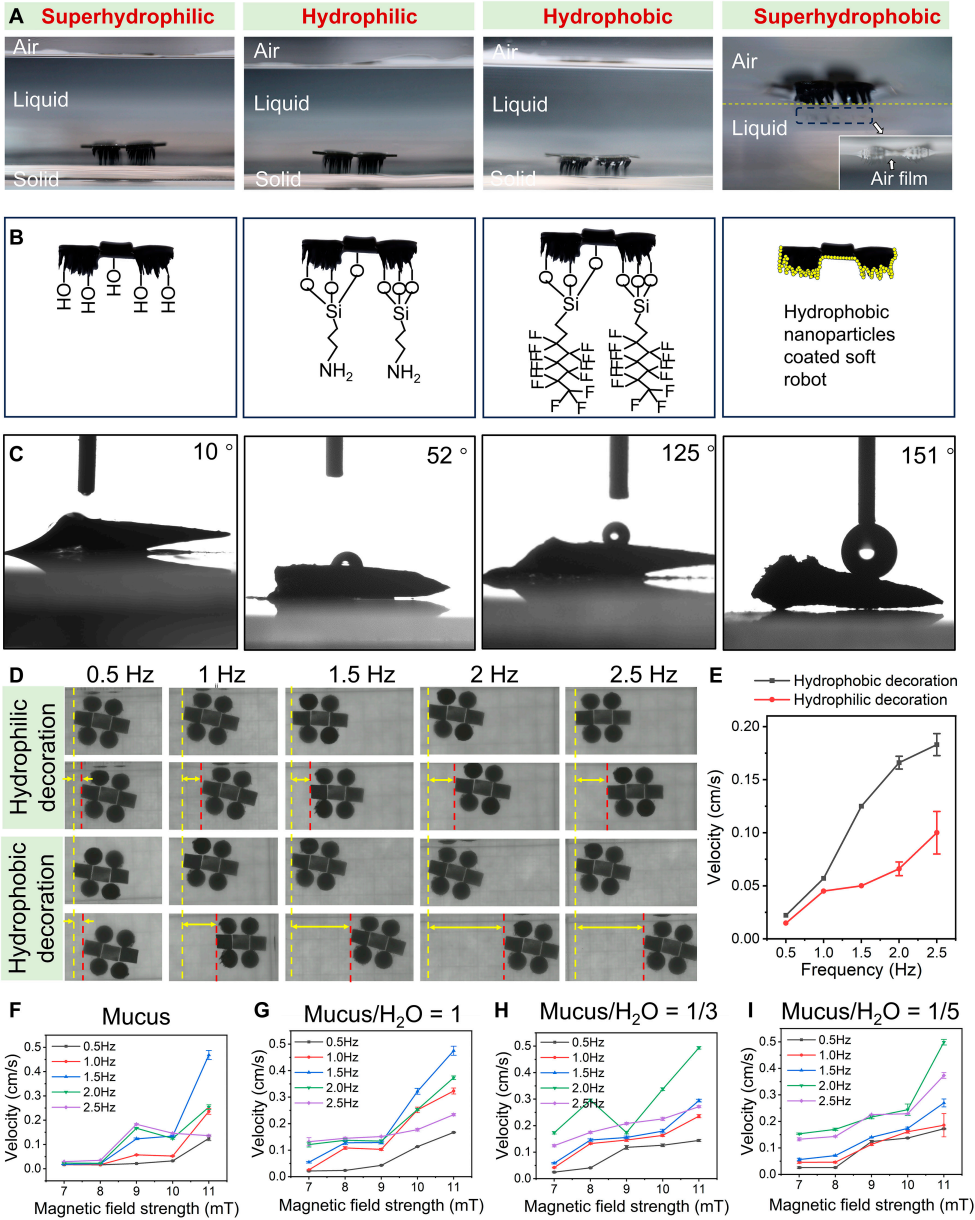
Motion behavior of the soft robots with hydrophilic and hydrophobic decoration. (A) Optical images showing the soft robots with superhydrophilic, hydrophilic, hydrophobic, and superhydrophobic modifications. (B) Schematic showing the surface coatings of superhydrophilic, hydrophilic, hydrophobic, and superhydrophobic soft robots. (C) Side view showing the wettabilities of the 4 kinds of robots. (D) Snapshots showing the motion behavior of hydrophilically decorated soft robots and hydrophobically decorated soft robots. (E) Curve showing the relationship between the motion velocity and the magnetic field frequency. (F to I) Curve showing the relationship between the motion velocity and the magnetic field frequency of hydrophobic soft robots under mucus environment with different diluted ratios.

To investigate the relationship between robot surface wettability and its motion performance, we have fabricated 4 kinds of soft robots with the same structure, whereas their surfaces were grafted with different molecules to endow the robots with different wettabilities [47]. As shown in Fig. 3A to C, while the surface of the soft robots is grafted with hydroxyl, amino, fluoroalkyl, and hydrophobic silica nanoparticles, we can obtain 4 kinds of soft robots with their surface contact angle approximately 10°, 52°, 125°, and 151°, respectively. These 4 functionalized soft robots exactly cover 4 categories of wetting, i.e., superhydrophilic, hydrophilic, hydrophobic, and superhydrophobic robots. As shown in Fig. 3A, while a superhydrophobic soft robot is placed on water surface, large amount of air will be trapped inside the nano- and microstructures of the robot, triggering the floating of the robot on water surface. It is unsuitable to select superhydrophobic robots as the payload carriers since they can hardly contact the lesion site to perform microsurgery or drug delivery process. For the other kinds of robots, we quantitatively compared the locomotion performance of the hydrophobic soft robots and the hydrophilic soft robots with the magnetic field parameters remaining in the same condition in a non-Newtonian liquid environment. Here, we select the mucus as the non-Newtonian liquid because it abundantly exists in the GI tract. One can observe from Fig. 3D that the locomotion distance of hydrophobic robots is much larger than that of hydrophilic robots (Fig. S11; field strength is 9 mT, motion time is 10 s). As the frequency of the magnetic field increases, the distance difference increases accordingly. From the relationship between motion speed and field frequency, one can observe that the motion speed is comparable for hydrophilic and hydrophobic robots, while the field frequency is lower than 1 Hz. However, as the frequency increases, the motion speed of hydrophobic robots shows a substantial improvement (2 times when the frequency is larger than 2 Hz) than that of hydrophilic ones (Fig. 3E and Movie S2). The highly increased motion performance of hydrophobic robots may be due to the low adhesion force between the surface hydrophobic molecules and water molecules. Moreover, the correlation between the velocity of the robot’s motion and the frequency (*f*) of the magnetic field is illustrated in Fig. S12. It is discernible that the velocity of the robot reaches its maximum at a frequency range specific to the robot, approximately between 2 and 4 Hz. Beyond *f* = 4 Hz, the crawling speed exhibits an inverse relationship with the frequency, indicative of the occurrence of an “out-of-step” phenomenon. This phenomenon manifests as the robot’s inability to promptly react to variations in the magnetic field, resulting in a diminished driving force and consequently influencing the robot’s movement speed. These observations collectively suggest an approximate step-out frequency for the robot within the range of 2 to 4 Hz. The interaction between the water and robot surface is highly dependent on the surface wettability and structure of the robot. Water comprises polar triatomic molecules with a bond angle of 104.5°. Surfaces with a strong polarity favor the capture of water molecules, whereas surfaces featuring nonpolar surface termination favor adverse effects [44]. Robot surface polarity is strongly associated with the surface terminal groups. Fluoroalkyl on the hydrophobic robots is nonpolar surface termination, and the weak interaction between nonpolar fluoroalkyl and polar water molecules facilitates the drag reduction during the robot motion (Fig. S13). We further investigated the motion performance of the hydrophobic soft robots under various liquid environments with different viscosities. We purchased fresh pig stomachs from the market and scraped them to obtain mucus, which was diluted to varying degrees with deionized water (Fig. S14). As shown in Fig. 3F to I, the velocity of the robot increases with magnetic field strength at all viscosities. The velocity of the robot increases with frequency until it reaches the maximum, and then the motion speed decreases with the further increase of the frequency. By comparison of Fig. 3F to I, one can observe that the step-out frequency increases with the continuous dilution of the mucus. The above results verified that hydrophobic modification of a robot will endow the robot with reduced interfacial friction, and the proposed LFSRs show exceptional motion capability under liquid environments with various viscosities.

### Improved motion performance by assembly of soft cones

**Fig. 4.**
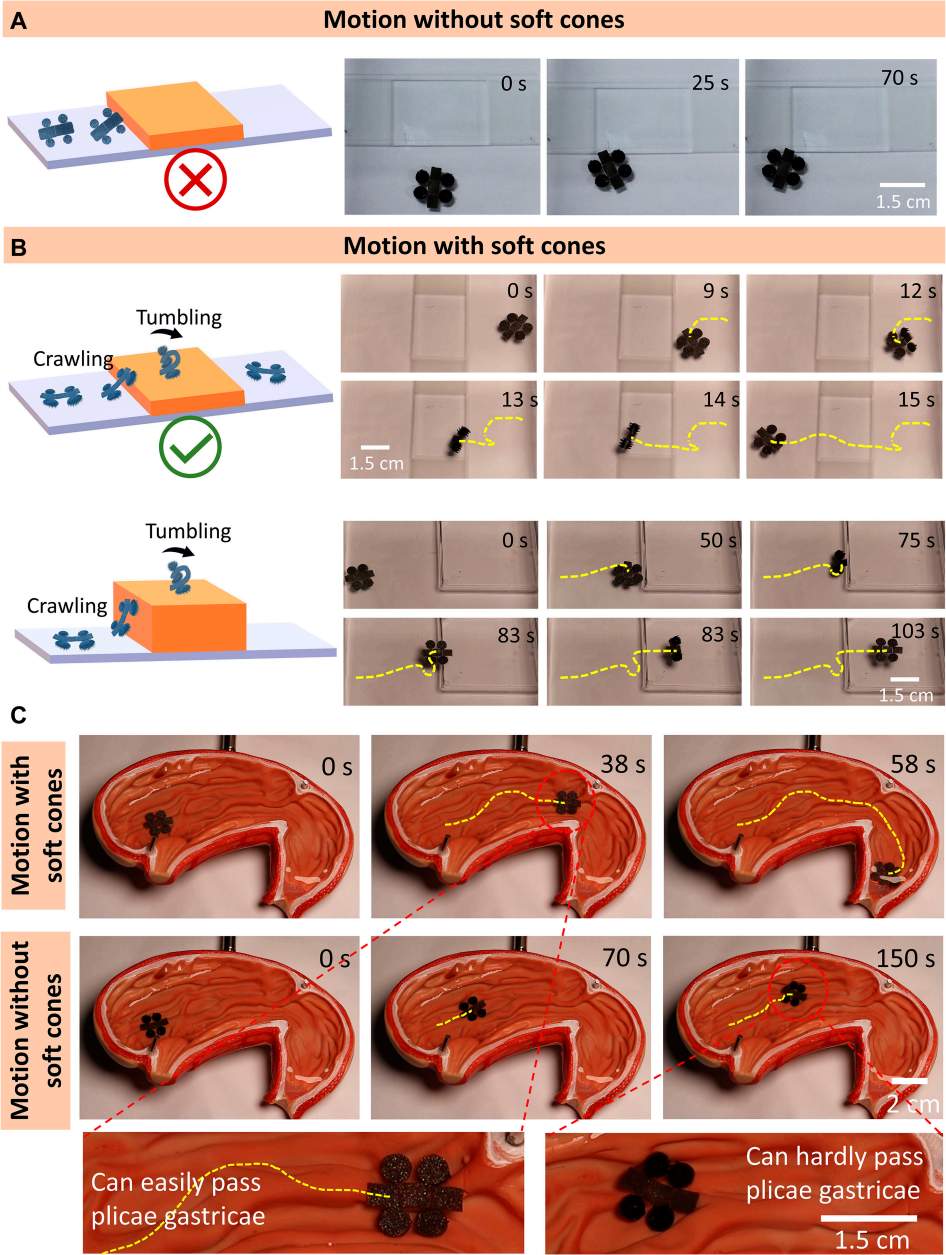
Motion performance of the LFSRs with soft cones and without cone structures. (A) Motion performance of the LFSRs without any cone structures. (B) Motion performance of the LFSRs with multiple soft cones (field strength is 30 mT, frequency is 2 Hz). (C) Magnetic navigation of LFSRs with and without multiple soft cones in a stomach phantom that is full of randomly distributed plicae gastricae (field strength is 30 mT, frequency is 2 Hz).

Apart from the surface wettability, motion performance is highly relevant with the robot structure. Natural animals and insects have developed an efficient method to reduce locomotion resistance by the evolution of multi-legged structures to lift the animal/insect’s body from the ground. The multi-legged structures endow the animals/insects with a higher degree of freedom in locomotion (obstacles can be overcome) and lower energy cost to overcome the interfacial friction. Inspired by legged living beings, development of multi-legged robots should be an efficient solution to overcome the complex terrains in the GI tract with a substantially reduced surface adhesion. Here, the soft cone-like structures are densely assembled on the foot part of the soft robot pre-encoded with specific magnetic orientation. Then, the developed soft robots both possess the magnetic encoding capability that facilitates programmable locomotion and a high degree of terrain adaptability to GI tract. To verify the terrain adaptability of our proposed robots, the motion performance of the same structured robots with multiple soft cones and without multiple soft cones is compared. As shown in Fig. 4A, for the soft robots without multiple soft cones, they can hardly pass through obstacles with a height of approximately 2 mm. For soft robots with multiple soft cones, they can curl and roll along their short axis while they encounter obstacles with a certain height (Fig. 4B). The robot with soft cones can smoothly pass through obstacles with a height of 2 mm by alternating their motion modes from crawling to tumbling (curling and rolling). During the crawling motion process, the robot’s arms dominate and bear alternating magnetization. The arms alternately move forward with the legs kicking the ground throughout the crawling. The robot moves forward with crossing some tiny obstacles. When the height of the obstacle exceeds the thickness of the robot itself (about 3 mm), the robot can cling to the edge of the obstacle through the cone structure of the feet to change the position of the body in the magnetic field, causing the robot’s body to curl under the asymmetric magnetic force. Until a critical point is reached, the robot loses balance due to excessive bending and begins to roll. When the robot crosses the obstacle, the direction of the magnetic field is adjusted so that the robot’s body bears again a uniform magnetic field force and expands under its own gravity. The robot resumes crawling motion. When continuing to increase the height of the obstacle, the curling and rolling become harder accordingly. The LFSRs with a body length of 1.5 cm are demonstrated to be capable of passing through an obstacle with a height of up to 6 mm (Fig. 4B and Movie S3), close to half of the robot’s body length. The LFSRs with soft cones are also verified to exhibit on-demand deformation of each module, arbitrary posture adjustment, and coordinated movement of modules (Movie S4). To further verify the excellent mobility of the proposed robots in GI tract, we performed the magnetic navigation experiment in a stomach phantom that is full of randomly distributed wrinkles (plicae gastricae). The soft robots with multiple soft cones can pass these barriers with ease, whereas the magnetic soft robots without soft cones are difficult to navigate with the same magnetic fields (Fig. 4C and Movie S5). Therefore, the LFSRs with multiple cones exhibit excellent maneuverability to travel across barriers on wrinkled surfaces, endowing significant potential for targeted delivery in non-Newtonian liquid environments.

### Magnetic field-coupled stiffness enhancement for mechanical biofilm destruction

Biofilms consist of diverse communities of bacteria enclosed within a self-produced polymeric matrix called exopolysaccharides (EPSs), which function as a survival mechanism aiding bacteria in adapting to and defending against harsh environments, antibiotics, and the immune systems of host organisms [48]. Various kinds of pathological biofilms may be generated in GI tract. For example, *Helicobacter pylori* biofilms have been widely observed in the gastric mucosa, and they can cause profound hypochlorhydria and activate pro-inflammatory pathways that are involved in further development of mucosal pathology [49]. Numerous approaches have been proposed to establish noninvasive treatment methods for biofilm formation and explored potential strategies such as broad-spectrum antibiotics for eliminating them and treating clinical bacterial infections. However, they exhibit limited antibacterial performance as biofilms exhibit reduced susceptibility to antibiotics compared to planktonic bacteria and can develop antibiotic resistance. To treat the bacteria-caused infection, novel surgical interventions should be developed to eradicate the biofilm.

**Fig. 5.**
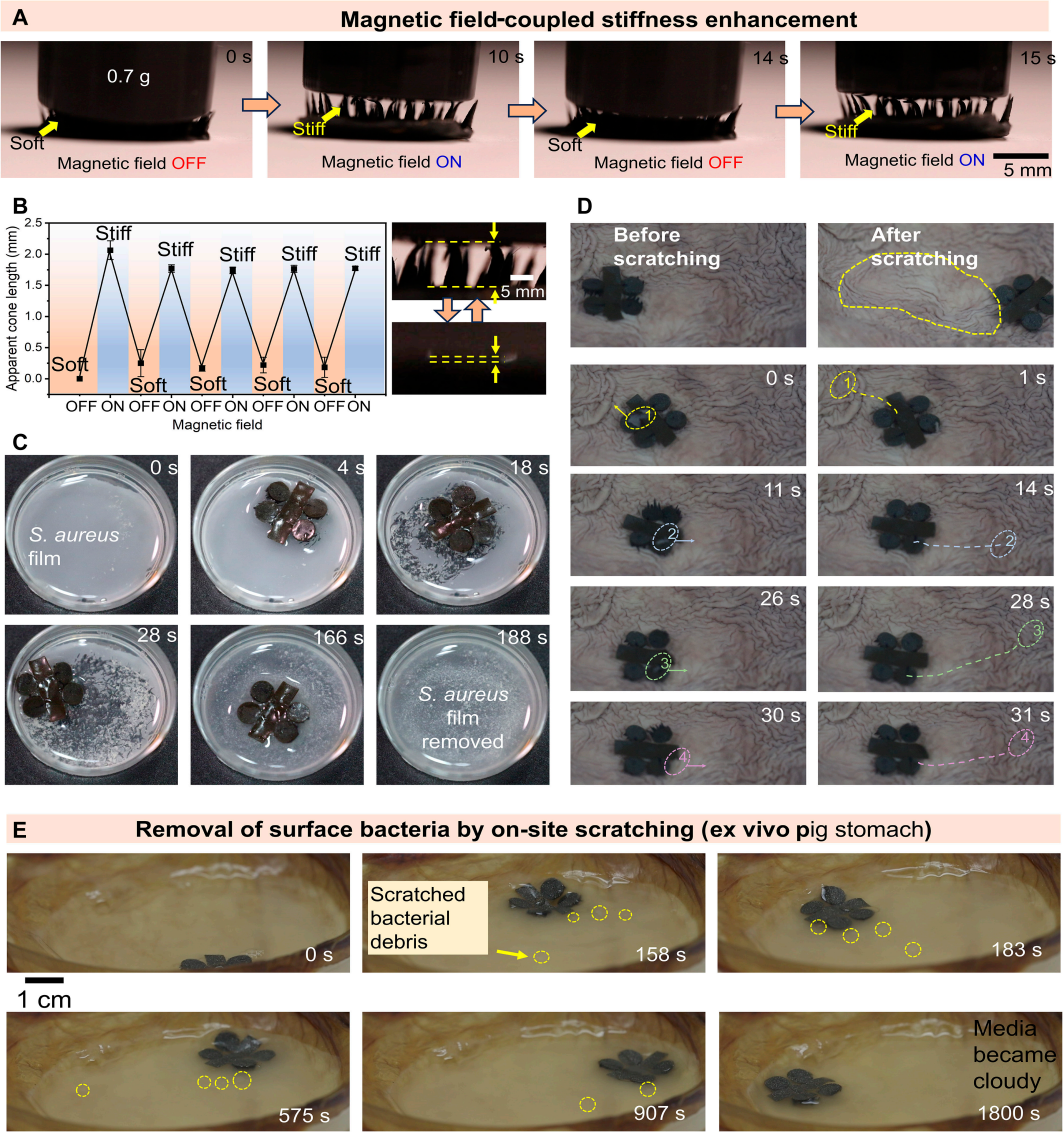
Magnetic field-coupled stiffness enhancement for mechanical biofilm destruction. (A) Magnetic field-coupled stiffness enhancement of the cone structures of the LFSRs. (B) Reversible conversion of soft state and rigid state of the cone structures of the LFSRs. (C) Magnetically induced hardening of the cone structures of the LFSRs for efficient removal of the bacterial film. (D) Controlled cleaning up of the debris of food and necrotic cells at the gap between stomach folds. (E) Controlled removal of the surface bacterial films by using an ex vivo GI tract model with *S. aureus* bacterial film infections.

Mechanical scratching is a straightforward but efficient method to destroy the polymeric matrix that existed in the biofilm. To endow the LFSRs with scratching capability, we enhance the stiffness by using the magnetic field. The mechanical properties of the soft cone structures on the LFSRs can be adjusted by using an external magnetic field. As intuitively shown in Fig. 5A (Movie S6), the mechanical properties of the multiple cones could be reversibly modulated by an external magnetic field, between soft and stiff. While the magnetic field is switched off, the multiple cones are soft and can be easily pushed down by an object with a mass of 0.7 g. While the magnetic field is switched on with a field strength of approximately 0.2 T, the multiple cones switch to a stiff state and the object can be lifted up due to the magnetically induced hardening. The magnetic particles inside the soft robots tend to align with a more dense and well-distributed microstructure, significantly increasing the mechanical stiffness. When the field was removed, the stiffness of cone structures suddenly disappeared, and the cone structures came back to soft characteristics The conversion between soft state and rigid state is revisable as demonstrated by Fig. 5B. Through the revisable control of the cone stiffness, the LFSRs are capable of performing on-demand scratching of the surface bacterial films with ease as demonstrated in Fig. 5C (*Staphylococcus aureus*; Movie S6) and Fig. S15 (*Escherichia coli*). The biofilm of *S. aureus* on a culture dish can be rapidly removed within 3 min by the robot with a magnetic field of 100 mT and a frequency of 3 Hz. The rigid feature of the cone structure under magnetic field can be well utilized to clean up the debris of food and necrotic cells at the gap between stomach folds (Fig. 5D). Moreover, we have also established an ex vivo GI tract model to simulate bacterial film infections on the surface of the GI tract, by growing *S. aureus* on the inner wall of an ex vivo GI tract model of pig (Fig. 5E and Movie S6). We found that the developed LFSR could efficiently remove the surface *S. aureus* film through magnetically hardened robot cone structures (field strength is 100 mT, field frequency is 3 Hz), similar to the sweeping process of a sweeping robot.

### Tracking of the low-friction robots in GI tract

To verify the potential use of the LFSRs for targeted bacterial infection treatment inside the body, we developed clinical imaging-guided actuation platforms that integrate the clinical imaging modalities, i.e., US imaging and x-ray imaging, together with a robot arm equipped magnetic actuation setup.

**Fig. 6.**
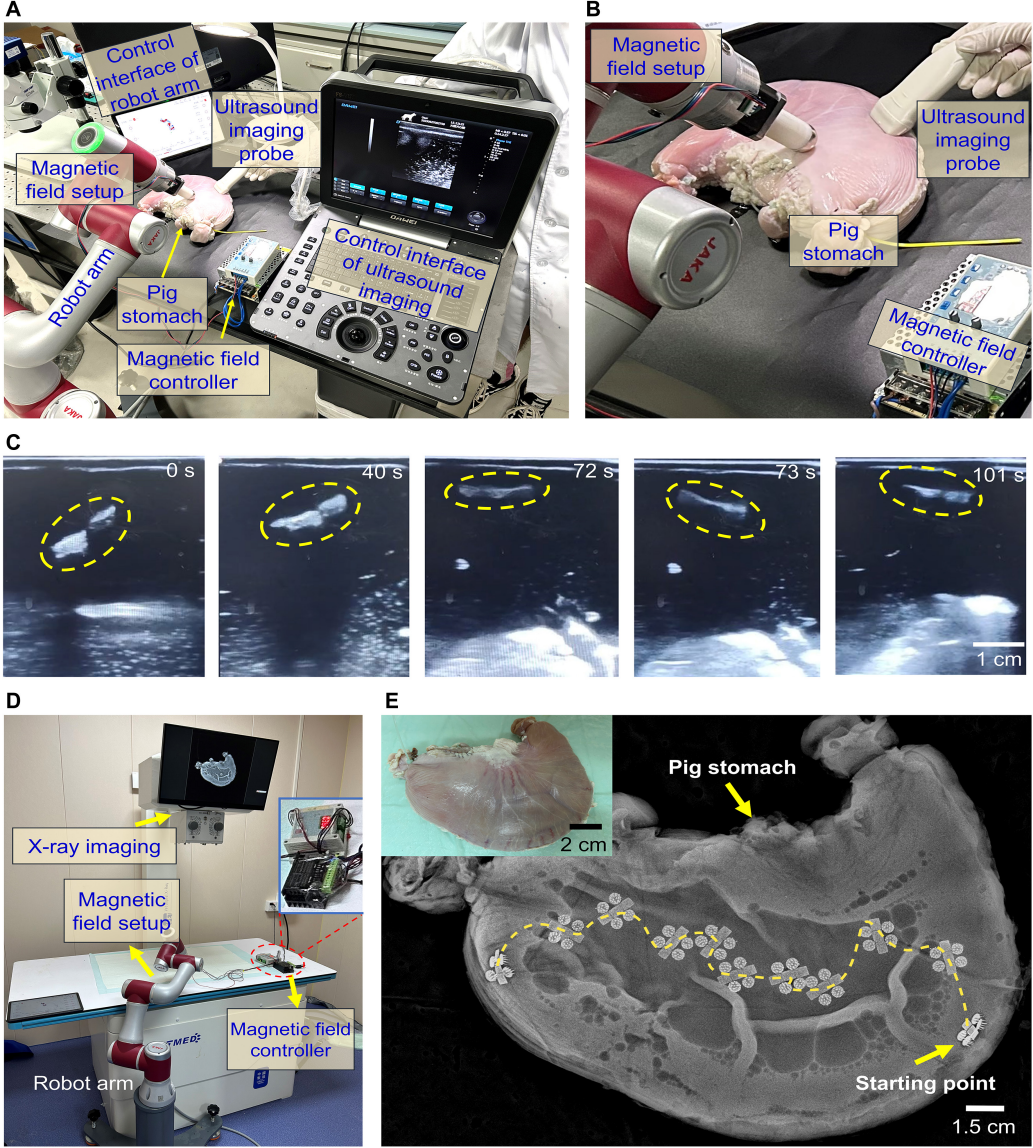
Controlled navigation and localization of an LFSR in pig stomach. (A) Overview of the experimental setup, including robot arm for magnetic field control, US imaging system, and a pig stomach. (B) Enlarged view showing the magnetic field setup assembled on the terminal of a robot arm, US imaging probe, and pig stomach. (C) Magnetically controlled locomotion (field strength is 120 mT, frequency is 1 Hz) of the LFSR in pig stomach with real-time tracking by US imaging. (D) Overview of the integrated experimental setup, including x-ray imaging system and magnetic field generator equipped at the terminal of a robot arm. (E) Successive images showing the magnetic field-guided motion trajectory of an LFSR in pig stomach (ex vivo).

Due to the deep penetration of the treatment site into soft tissues within the abdominal cavity, US imaging can be used for real-time localization of the robot, especially in environments filled with fluid. Figure 6A and Fig. S16 demonstrate the experimental setup for the driving process of the robot in an ex vivo pig stomach under US imaging guidance. The ex vivo pig stomach is placed under the driving unit, which consists of a robotic arm and an electric motor. Subsequently, an imaging unit, a US probe, is placed on the surface of the pig stomach to track the robot’s movement in real time. Figure 6B presents a close-range optical image of the pig stomach within the operating system. Figure 6C (Movie S7) illustrates the US tracking of a single robot under programmable navigation in a magnetic field. The results indicate that the robot’s motion within the ex vivo pig stomach can be clearly visualized with bright contrast using US imaging technology. This experiment demonstrates the feasibility of real-time US imaging-guided robot tracking and its potential application in bacterial infection therapy.

When the treatment area is deep inside the body and obstructed by bones and air layers, x-ray can be considered as one of the methods to address these situations. It has become a widely used clinical imaging device in hospitals worldwide. Although x-ray has limitations in providing detailed assessment of medical conditions, it is suitable for locating low-attenuation magnetic microrobots in any part of the body, especially in deep tissues. Therefore, x-ray can also be used as a means of tracking robots. Figure S17 gives the mechanism of x-ray imaging applied for the tracking of microrobots inside body. Figure 6D shows the overall experimental setup for imaging and tracking robots using x-ray, including a driving system consisting of a mechanical arm and electric motor, and an x-ray imaging system. Figure 6E and Fig. S17 (Movie S7) demonstrate the movement trajectory of the robot inside the isolated pig stomach under x-ray tracking imaging, with the robot’s feet clearly visible in the x-ray imaging. These results verify the feasibility of using x-ray for tracking robots inside GI tract.

### Magnetocaloric effect-based bacterial infection treatment

**Fig. 7.**
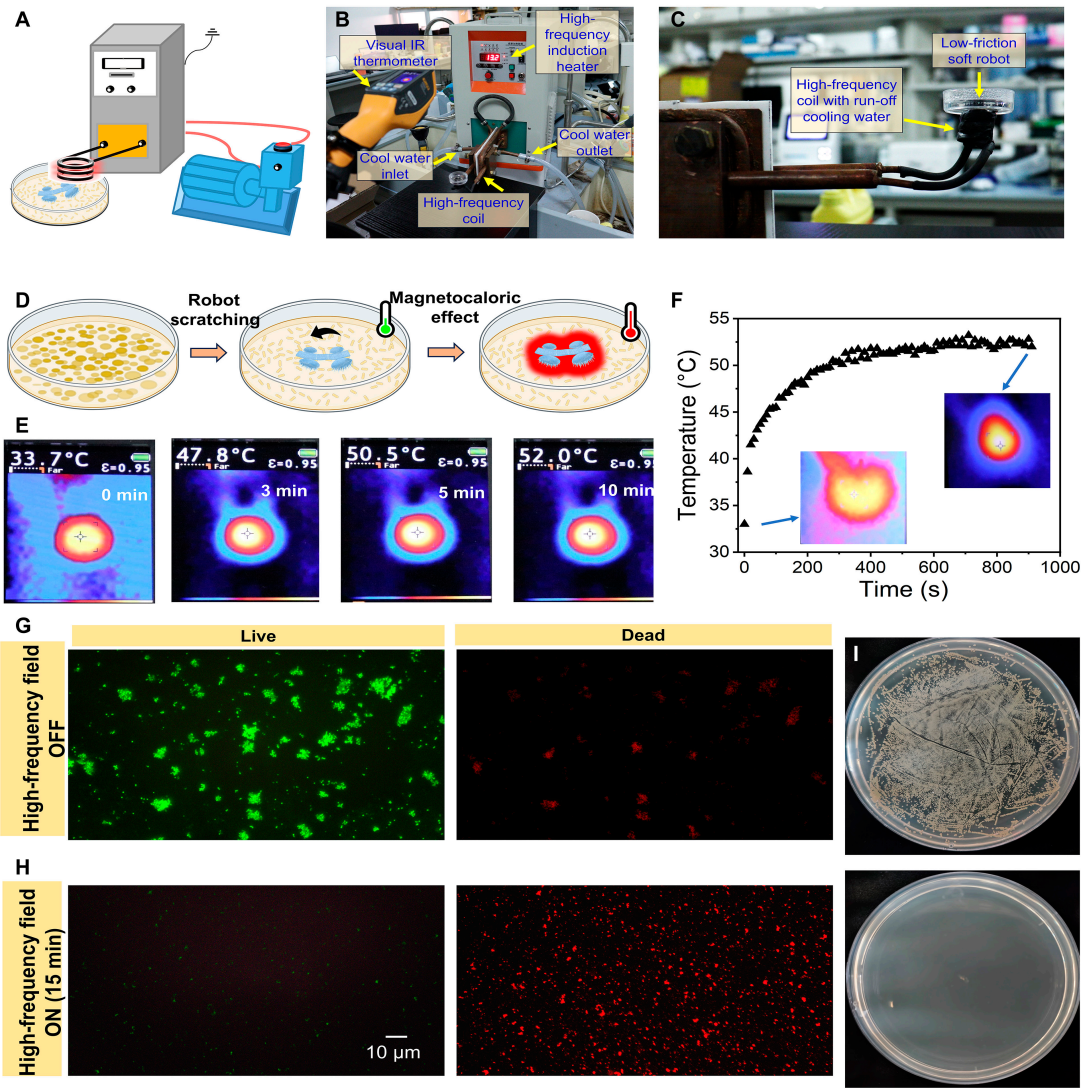
On-demand treatment of bacterial infection by using high-frequency AMF. (A) Schematic showing the setup of high-frequency AMF with circulating water cooling. (B) Photograph of the high-frequency AMF setup. (C) Photograph of the heating coil of the high-frequency AMF setup. (D) Schematic showing the bacterial infection treatment process by magnetocaloric effect. (E) Visual IR thermometer images showing that the temperature raise accumulated over time. (F) Curve showing the relationship between temperature and time. (G) Fluorescent images showing the *S. aureus* after the magnetic field-induced scratching process without applying high-frequency AMF. (H) Fluorescent images showing the *S. aureus* after the magnetic field-induced scratching process and magnetic hyperthermia by applying high-frequency AMF. (I) Photographs showing the bacterial recultivation result of the *S. aureus* without and with applying high-frequency AMF, respectively.

While the low-frequency magnetic field has been verified to be an efficient method to sweep out the bacterial films via magnetically hardening effect, a high-frequency AMF can be further applied to generate temperature raise for bacterial killing through magnetocaloric effect. The high-frequency AMF can cause the magnetization reversal process periodically. The magnetic particles inside the robot come back to their relaxed states periodically, and the thermal energy is continuously generated. Figure 7A shows the schematic of the high-frequency AMF generator that is mainly composed of a group of hollow copper coils with cooling water continuously flowing through copper coils. Figure 7B and C show the photographs of the treatment of bacterial infection by using LFSRs under high-frequency AMF (300 kHz). After the *S. aureus* film was scratched by low-frequency magnetic field, a high-frequency AMF with a frequency of 300 kHz is applied to further kill the bacteria (Fig. 7D). The temperature gradually raised up from room temperature, which was recorded by a visual infrared (IR) thermometer (Fig. 7E and Movie S8). The temperature was raised to 52 °C within 10 min and finally reached a plain stage (Fig. 7F). After 15 min of high-frequency AMF treatment, the bacteria were stained for live and dead bacteria inspection. As indicated by Fig. 7G, the *S. aureus* without treatment by high-frequency AMF remained alive with a few dead bacteria, which indicated that magnetic field-induced scratching process could destroy the bacterial films but cannot kill the bacteria. While the *S. aureus* solution is treated by high-frequency AMF, the fluorescent images in Fig. 7H clearly indicate that the bacteria are dead. Furthermore, the remaining *S. aureus* solution of both the above 2 groups is collected and the bacterial recultivation is performed. After 1-day incubation, a large number of bacteria colonies are observed for the control group without AMF (Fig. 7I). On the contrary, no obvious bacteria colonies appear in the sample group with a high-frequency AMF applied. These results demonstrated that the magnetic hyperthermia treatment of the bacteria infection by using our LFSRs enables the efficient eradication of the bacteria, and compared with the other kinds of clinical technologies (such as oral drug medication and surgical operation), the LFSRs show their merits in high spatial precision, less trauma, painless treatment, and virtually no damage to normal tissues (Table S1).

## Conclusion

In summary, we developed an LFSR that possesses substantially improved motion performance in GI tract with non-Newtonian liquid environment and plicae gastricae obstacles. The LFSR shows exceptional stretchability and softness akin to human tissue, thereby eliminating the potential for tissue damage during targeted delivery through the GI tract. The developed soft robots both possess the magnetic encoding capability that facilitates programmable locomotion and a high degree of terrain adaptability to GI tract. With the application of an external magnetic field, the soft robots can move in multiple modes, with a substantially reduced drag and improved motion velocity across the non-Newtonian liquids, such as mucus environment inside GI tract. The ultra-low surface friction and adhesion are generated by the rational design of the soft robots with multiple cone structures and hydrophobic decoration. Hydrophobic modification of a robot is found to generate reduced interfacial friction, endowing the LFSRs with exceptional motion capability under liquid environments with various viscosities. Notably, the magnetic particles inside the soft robots tend to align with a more dense and well-distributed microstructure under an external magnetic field, significantly increasing the mechanical stiffness. The robot stiffness can be reversibly controlled, enabling on-demand actuation and removal of bacterial films by on-site scratching with a low-frequency magnetic field.

Furthermore, the magnetocaloric effect can be utilized to heat up the LFSRs through a high-frequency AMF, enabling the thorough eradication of the bacteria. To perform targeted delivery of the robots inside body, we developed clinical imaging-guided actuation platforms that integrate the clinical imaging modalities, i.e., x-ray imaging and US imaging, together with a robot arm-equipped magnetic actuation setup. The robot’s motion trajectory, as well as the biofilm scratching, can be detected using clinical imaging-guided actuation platforms. The developed low-friction robots and the clinical imaging-guided actuation platforms show their high potential to perform bacterial infection therapy in various non-Newtonian liquid lumens. The magnetic field-responsive mechanically adaptive robots provide a new strategy to destroy the biofilm inside the lumens of human body but do not cause damage to the healthy tissues by timely control of robot stiffness through a remote magnetic field.

## Data Availability

All data are available in the manuscript or the Supplementary Materials.
