# Supplementary material for: Low-Friction Soft Robots for Targeted Bacterial Infection Treatment in Gastrointestinal Tract
Source: Cyborg Bionic Syst. 2024 Jul 5;5:0138. doi: 10.34133/cbsystems.0138 (PMC11223897; doi:10.34133/cbsystems.0138)
Supplement: Supplementary 1 — Figs. S1 to S17 Table S1 Movies S1 to S8 [file cbsystems.0138.f1.zip › Supplemental Material 20240505.pdf]

Supplemental Material

Low-friction soft robots for targeted bacterial infection treatment in gastrointestinal tract

Ben Wang[1,], Yunrui Chen[1], Zhicheng Ye[1], Haidong Yu[2], Kai Fung Chan[3,*], Tiantian Xu[4,5,*], Zhiguang Guo[6,7,*], Weimin Liu[7], Li Zhang[8,9]*

[1]College of Chemistry and Environmental Engineering, Shenzhen University, Shenzhen, 518060, China

[2]Guangxi Key Laboratory of Processing for Non-Ferrous Metals and Featured Materials, School of Resource, Environments and Materials, Guangxi University, Nanning 530004, China

[3]Chow Yuk Ho Technology Centre for Innovative Medicine, The Chinese University of Hong Kong, Shatin, New Territories, Hong Kong SAR, China

[4]Guangdong Provincial Key Lab of Robotics and Intelligent System, Shenzhen Institute of Advanced Technology, Chinese Academy of Sciences, Shenzhen 518055, China

[5]SIAT Branch, Shenzhen Institute of Artificial Intelligence and Robotics for Society, Shenzhen 518055, China

[6]Hubei Collaborative Innovation Centre for Advanced Organic Chemical Materials and Ministry of Education Key Laboratory for the Green Preparation and Application of Functional Materials, Hubei University, Wuhan 430062, PR China

[7]State Key Laboratory of Solid Lubrication, Lanzhou Institute of Chemical Physics, Chinese Academy of Science, Lanzhou 730000, China

[8]Department of Mechanical and Automation Engineering, The Chinese University of Hong Kong, Shatin, New Territories, Hong Kong SAR, China

[9]Multi-Scale Medical Robotics Center, Hong Kong Science Park, Shatin, New Territories, Hong Kong SAR, China

*Address correspondence to:

B.W.: benwang@szu.edu.cn; K.F.C.: kaifungchan@cuhk.edu.hk; T.T.X.: tt.xu@siat.ac.cn; Z.G.: zguo@licp.cas.cn

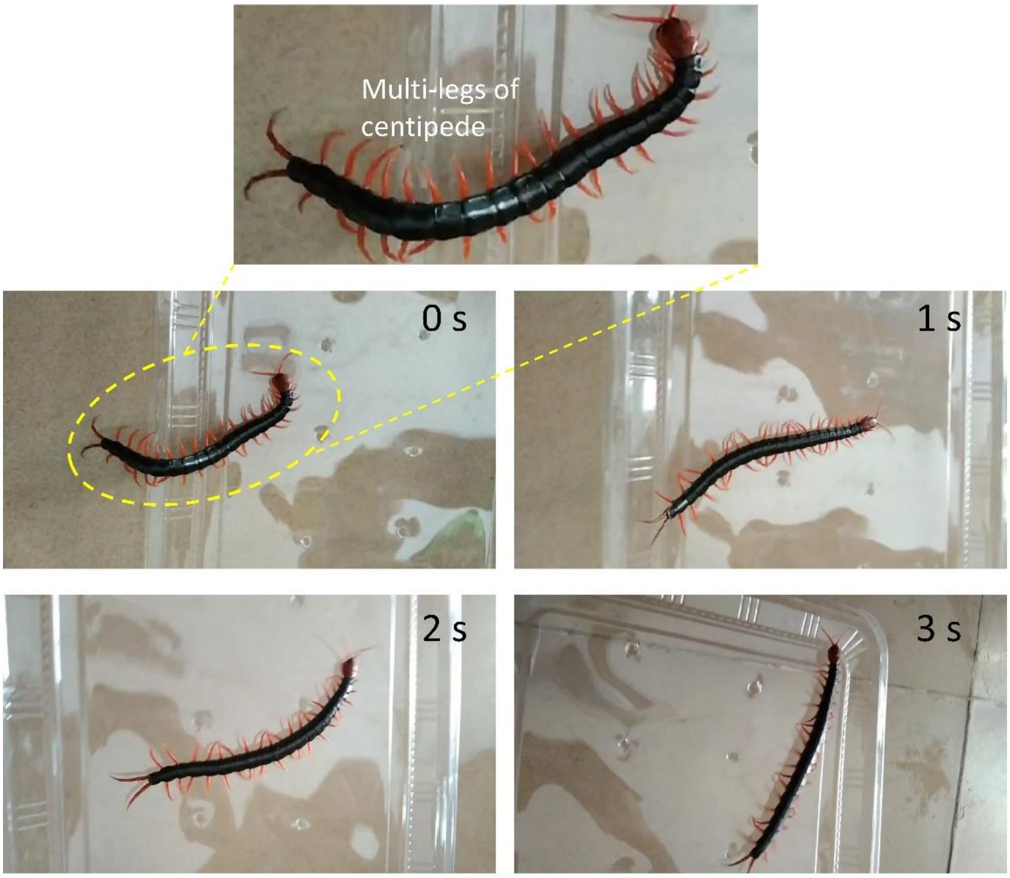

Figure S1. Centipede crawling----the locomotion inspiration source of low-friction soft robot.

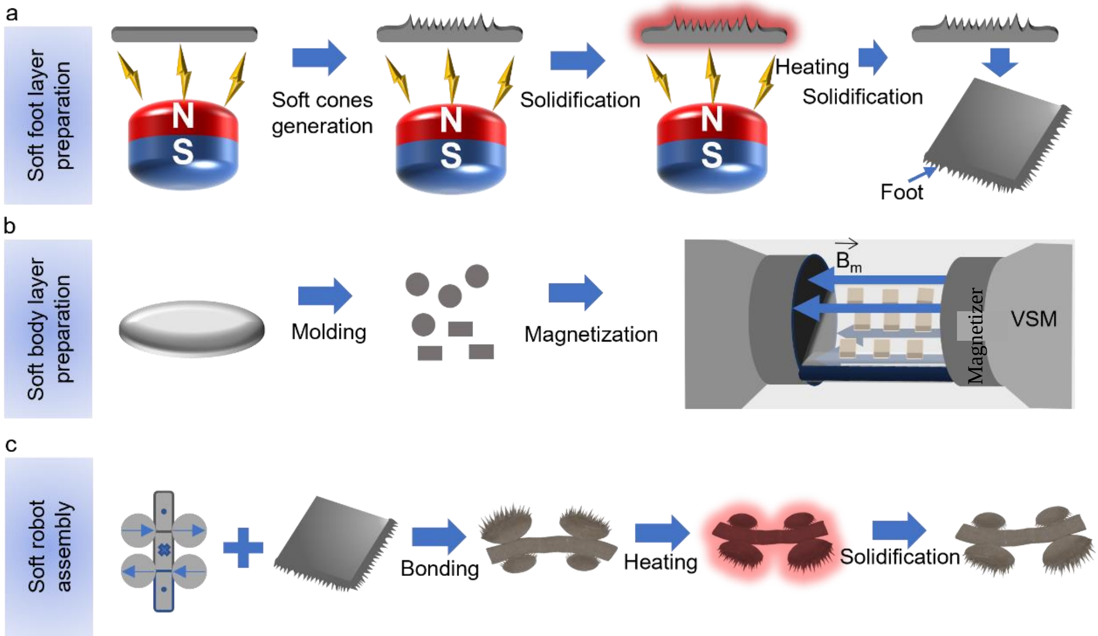

Figure S2. Schematic showing the fabrication procedures of the low-friction soft robots.

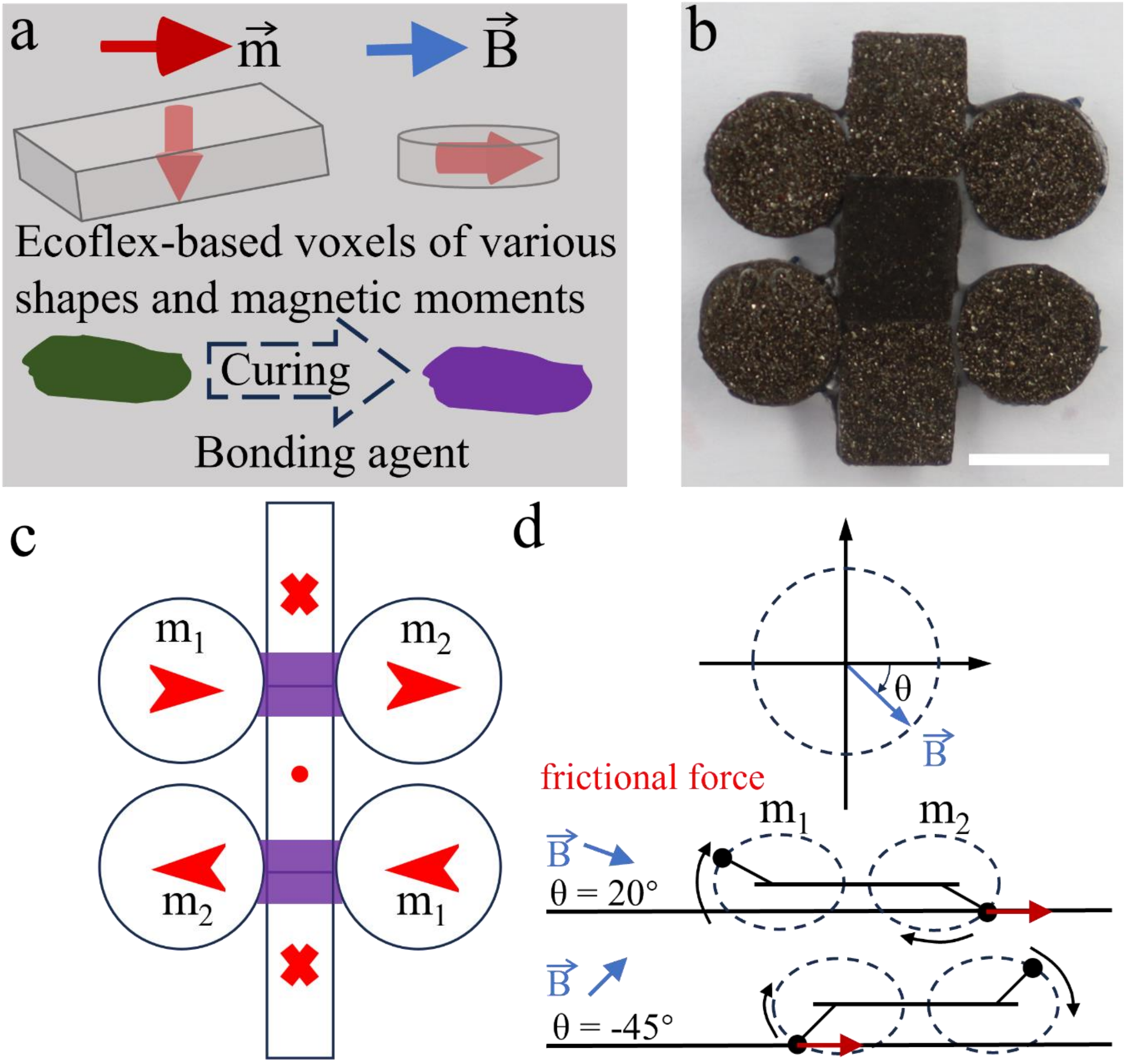

Figure S3. (a) Ecoflex-based voxels with different shapes and magnetization profiles $\vec{m}$ were integrated together by a bonding agent to build low-friction soft robots. Image (b) and magnetization profile (c) of a low-friction soft robot. Local magnetization is denoted by red arrows. When the legs labeled m_1 perform power strokes, the legs labeled m_2 perform recovery strokes, and vice versa. Scale bar, 5 mm. (d) Schematic representation of the gait from the side.

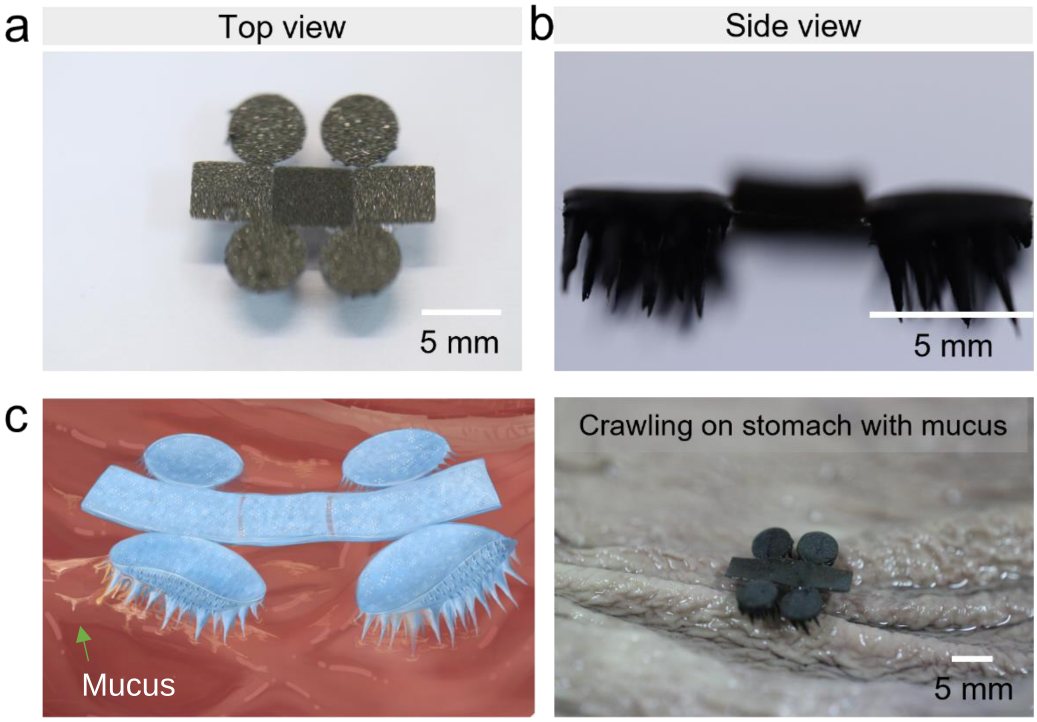

Figure S4. Top view (a) and side view (b) of the low-friction soft robot. (c) Schematic and optical image showing the crawling on GI tract with mucus of the low-friction soft robot.

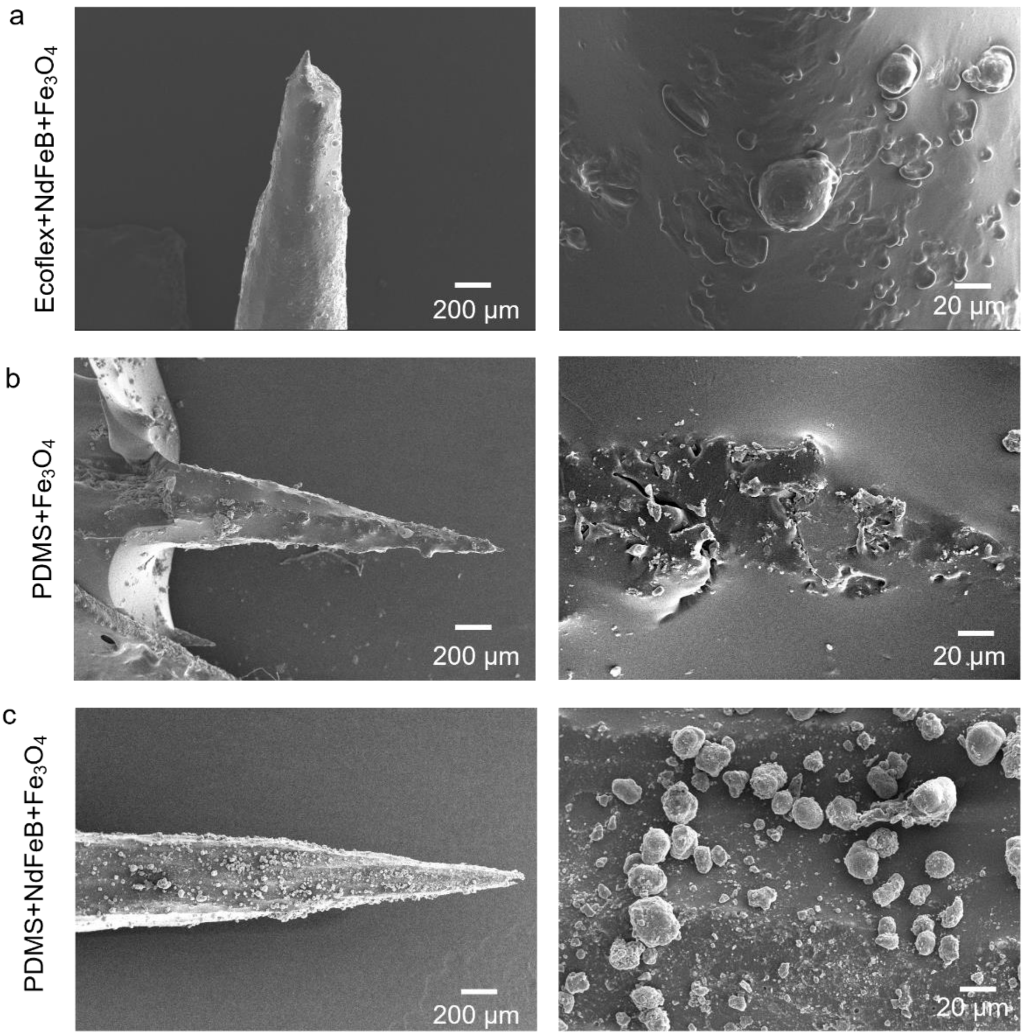

Figure S5. SEM images of the magnetic soft cones on the robot foot layer prepared using different materials.

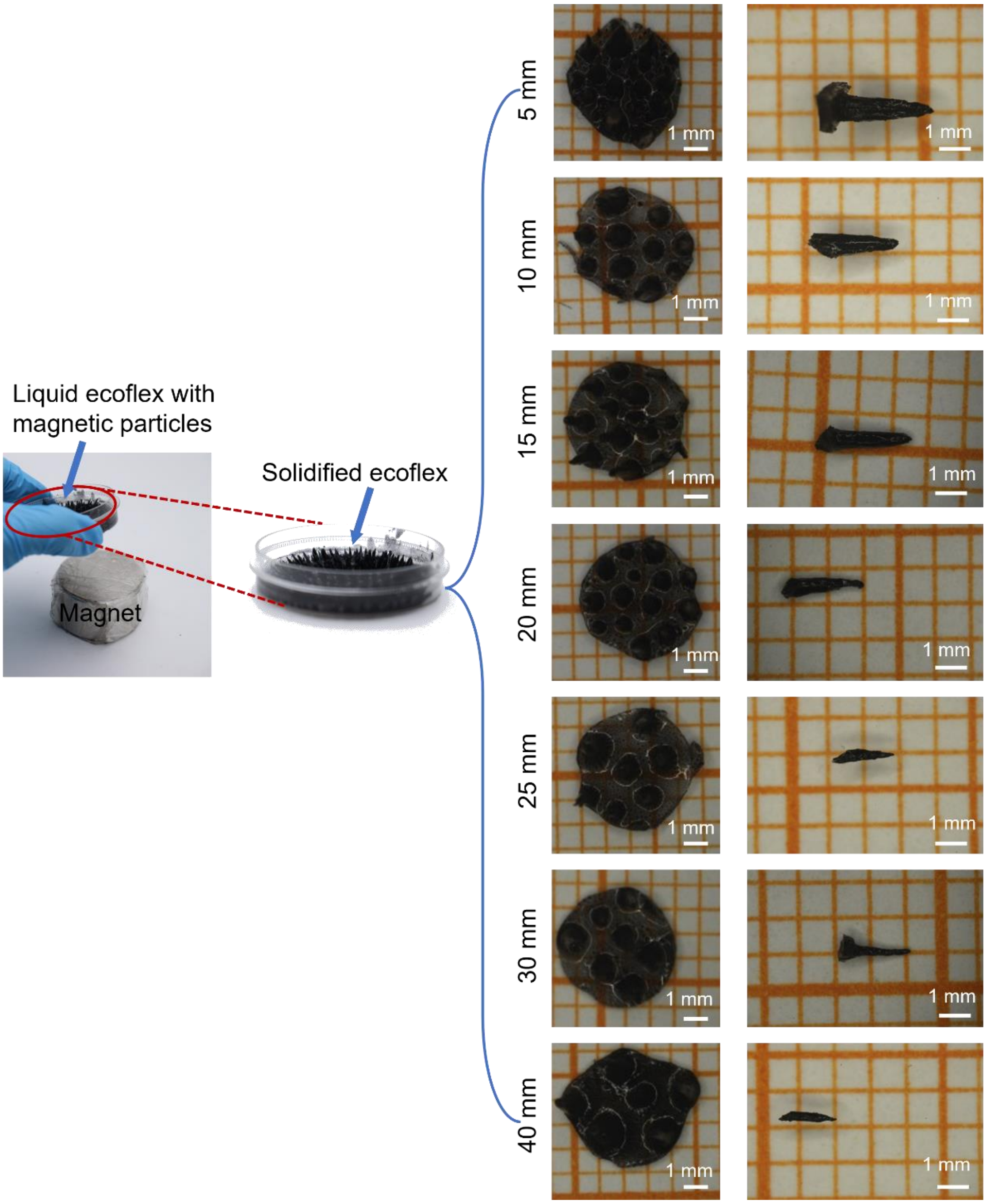

Figure S6. Cones of the low-friction soft robot of varying density and length depending on the distance of magnet and prepolymer (mixture of Fe_3O_4 particles and Ecoflex).

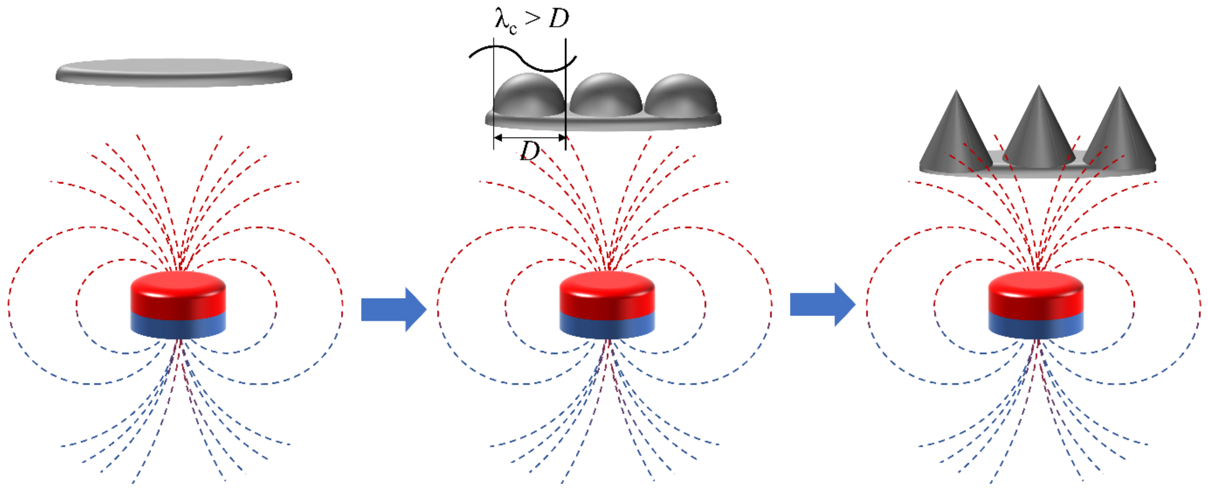

Figure S7. Schematic showing the splitting instability of magnetic prepolymer.

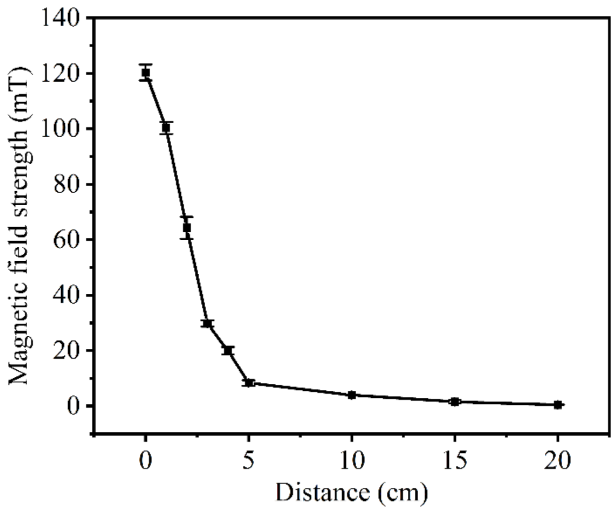

Figure S8. Dependence of the magnetic field strength of the permanent magnet used for the preparation of the cone structures.

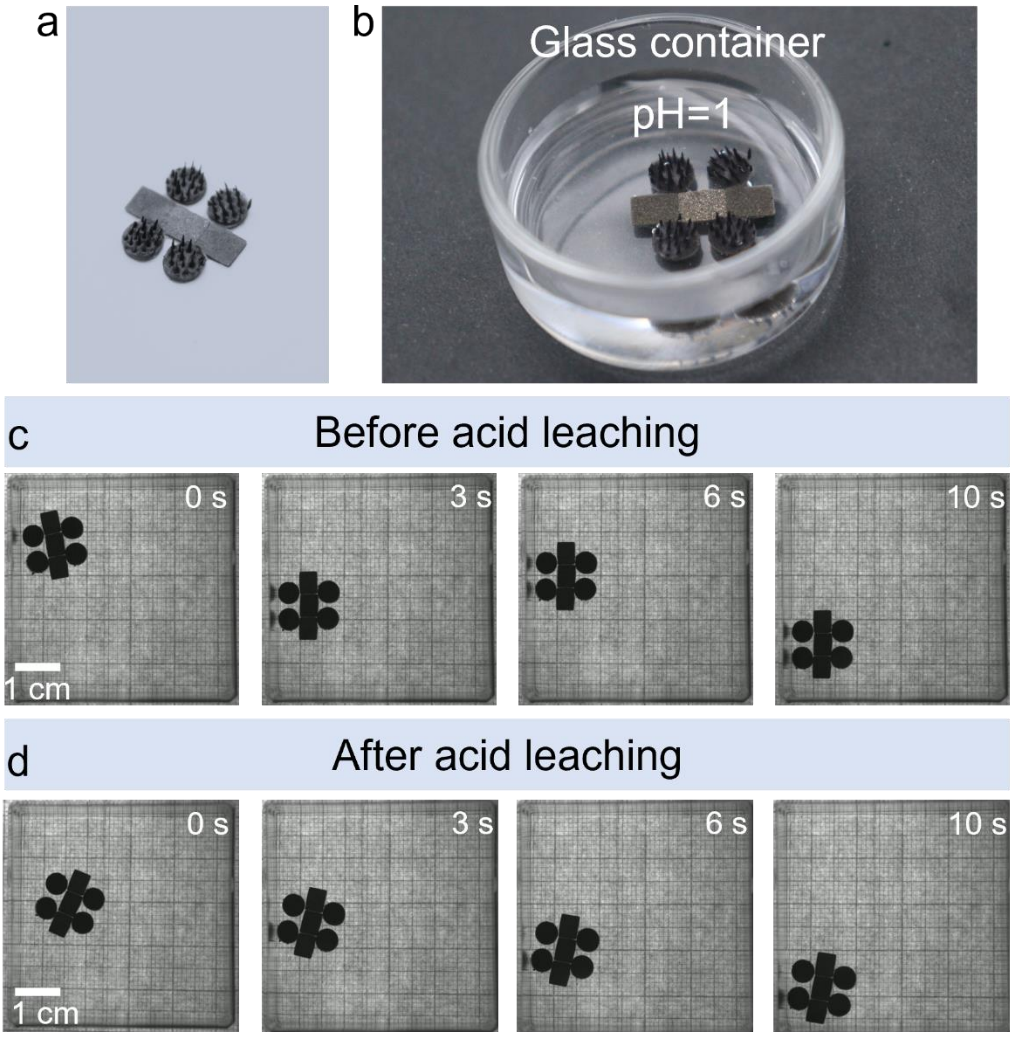

Figure S9. Optical image of a low-friction soft robot before (a) and after (b) immersion in acid (pH=1) solution for 6 h. Motion performance of the robot before (c) and after (d) acid immersion (frequency is 2 Hz, field strength is 9 mT).

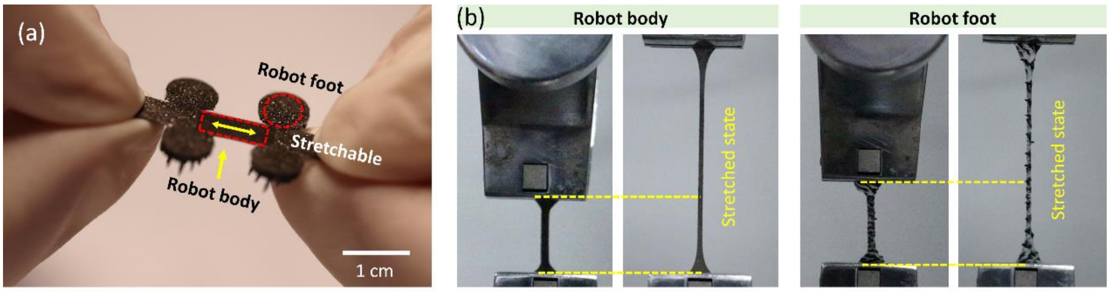

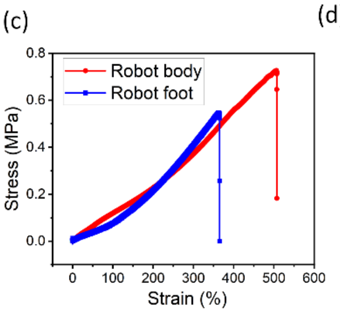

(d)

Sample	Elastic modulus(kPa)
Bone	1.5×10^4~2×10^4
Cartilage	1×10^3~1.5×10^3
Intestine	20~40
Heart	10~15
Brain	1~4
Bone marrow	0.5~1.5

Figure S10. Mechanical properties of the low-friction soft robots. (a) Photograph showing the stretchable soft robots, containing the robot body and robot feet. (b) Photograph showing the tensile test of the body part and foot part of the low-friction soft robots. (c) Stress-strain curve of the low-friction soft robot under the tensile machine. (d) Table of the elastic modulus of different human tissues and organs.

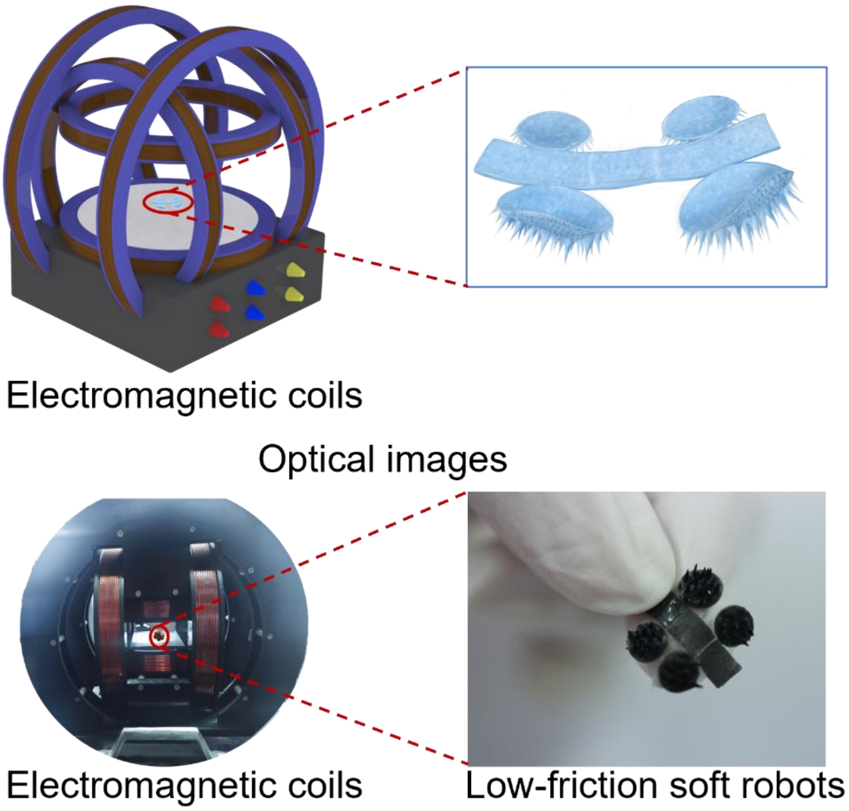

Figure S11. Helmholtz coils setup for controlled locomotion of the low-friction soft robot.

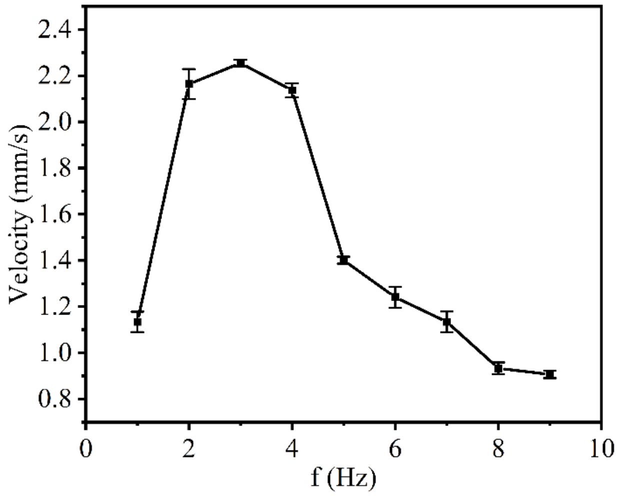

Figure S12. The motion performance of the hydrophobic robot at different frequencies (field strength is 9 mT).

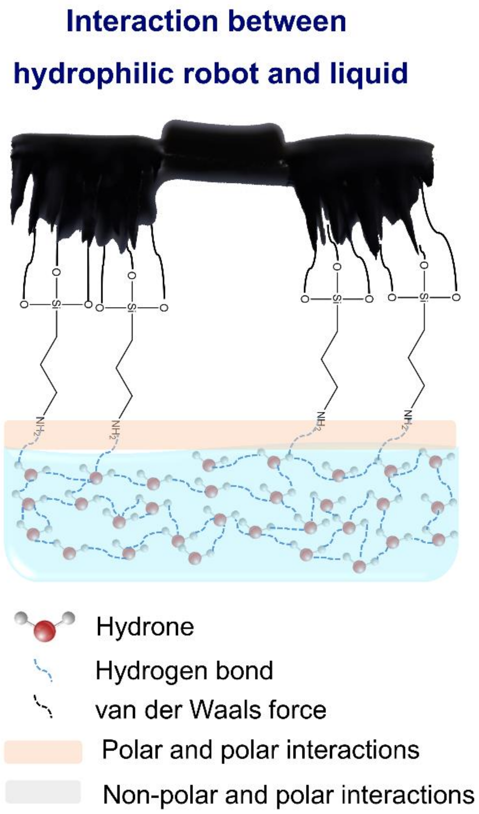

Figure S13. Schematic showing the interactions between hydrophilic robot, hydrophobic robot and the liquid molecules, reflecting weaker interaction between non-polar fluoroalkyl and polar water molecules.

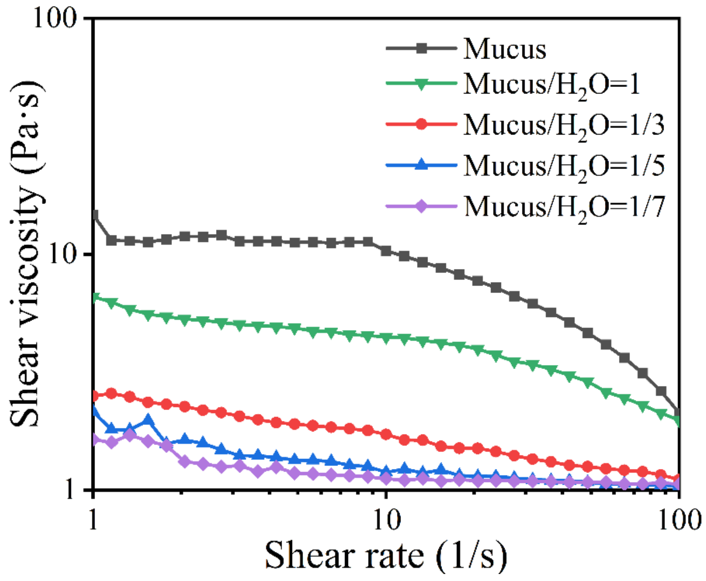

Figure S14. Relationship between the viscosity of the mucus and the applied shear rate under different dilution ratios.

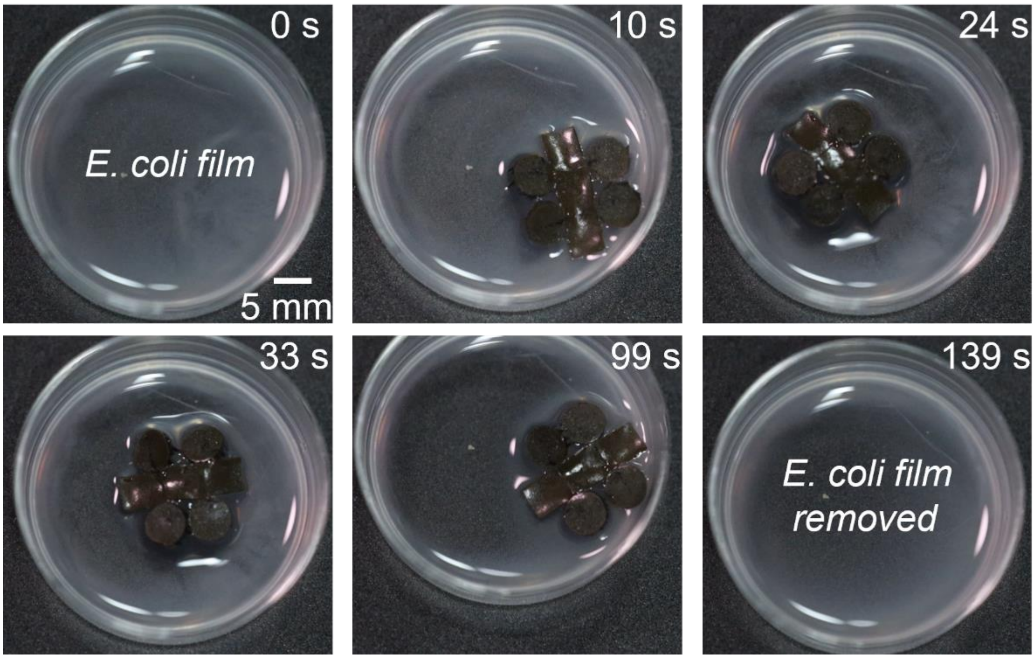

Figure S15. Magnetically induced hardening of the cone structures of the low-friction soft robots for efficient removal of the bacterial film of *E. coli*.

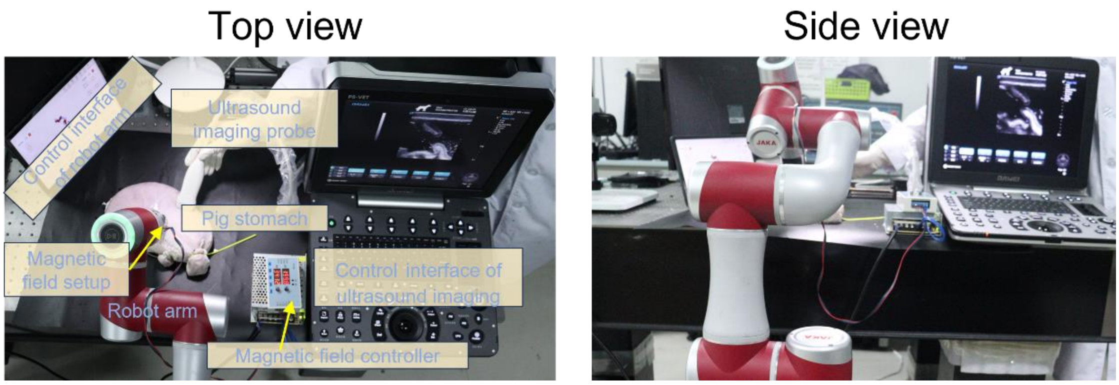

Figure S16. Top view and side view of the experimental setup for controlled locomotion in pig stomach with real-time localization by using US imaging, including robot arm for magnetic field control and position tracking system as well as the pig stomach.

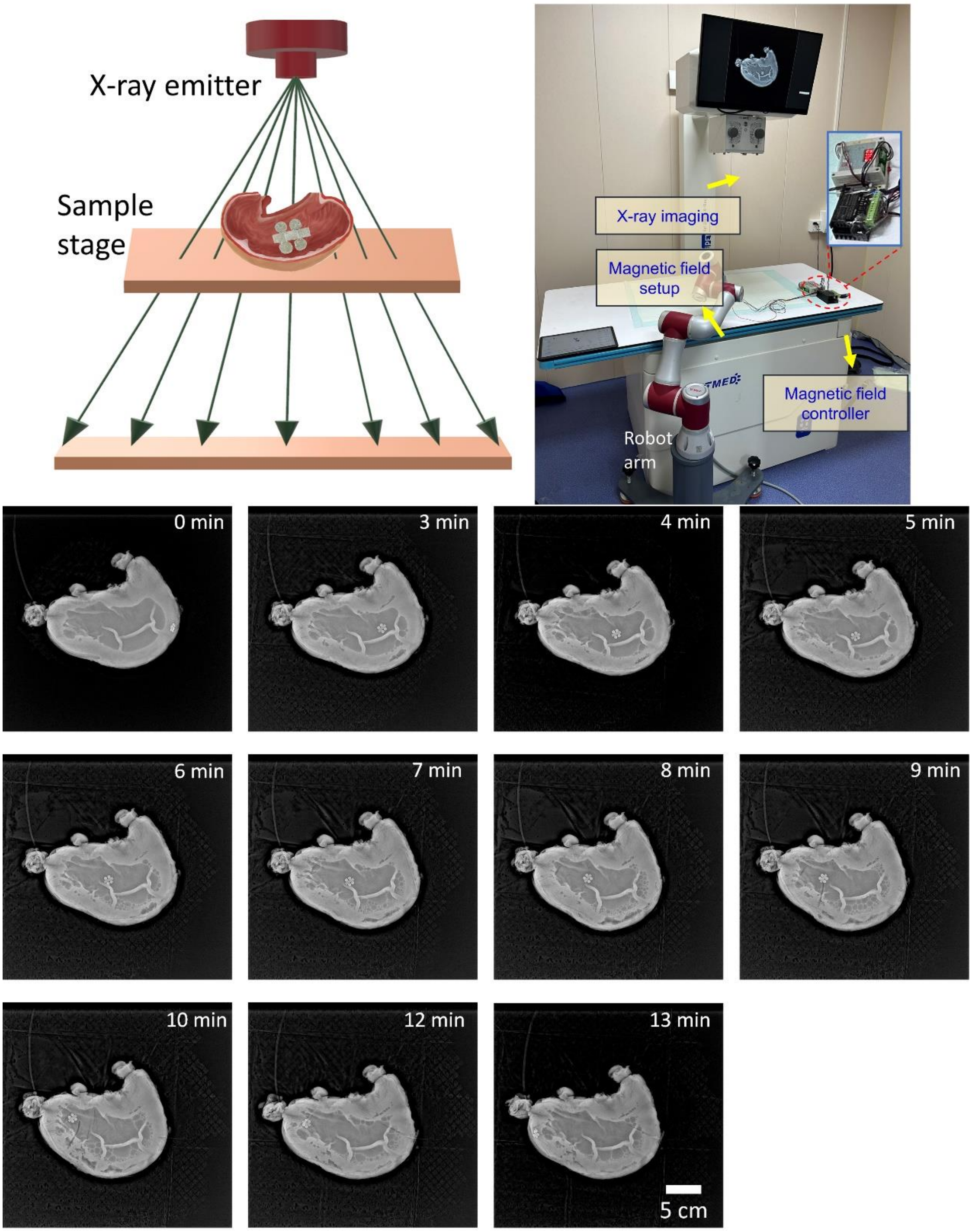

Figure S17. Controlled navigation and localization of a low-friction soft robot in pig stomach by X-ray imaging.

Table S1. Summary and comparison of LSFRs and other existing bacterial infection therapies.

Tool	Advantages	Limitations	Treatable Symptoms
Oral Drug Medication	Low trauma	Inaccurate treatment	GI tract related diseases
	Minimal body damage	Drug resistance	
	Easy operation	Significant side effects	
Surgical Operation	Quick and thorough treatment	High trauma	Excision of lesions of severe GI tract bacterial infection
	Limited further spread of infection	More body damage	
LFSRs	High spatial precision	Difficult operation	Full-term treatment of early and mid-stage diseases of GI tract
	Painless treatment		
	Virtually no damage		
